# Supplementary material for: Differential KEAP1/NRF2 mediated signaling widens the therapeutic window of redox-targeting drugs in SCLC therapy
Source: Nat Commun. 2026 Apr 12;17:3435. doi: 10.1038/s41467-026-71608-4 (PMC13076645; doi:10.1038/s41467-026-71608-4)
Supplement: Supplementary file 1 — Supplementary Information [file 41467_2026_71608_MOESM1_ESM.pdf]

Supplementary Information for:

Differential KEAP1/NRF2 mediated signaling widens the therapeutic window of redox-targeting drugs in SCLC therapy

Jana Samarin[1$], Hana Nůsková[1$], Piotr Fabrowski[1], Mona Malz[1], Eberhard Amtmann[1], Minerva J. Taeubert[1], Daniel Pastor-Flores[2,3], Daniel Kazdal[4,5], Roman Kurilov[6], Nicole de Vries[1], Hannelore Pink[1], Franziska Deis[1], Johanna Hummel-Eisenbeiss[1], Lisa Renz[1], Kamini Kaushal[7], Michael Morgen[1], Tobias P. Dick[2,3], Gerhard Hamilton[8], Martina Muckenthaler[9], Moritz Mall[10,11,12], Bryce Lim[10,11,12], Taishi Kanamaru[10,11,12], Glynis Klinke[13], Martin L. Sos[14,15,16,17], Julia Frede[14,15,18], Aubry K. Miller[1,19], Hamed Alborzinia[7] and Nikolas Gunkel[1, 19,*]

1 German Cancer Research Center (DKFZ) Heidelberg, Research Group Cancer Drug Development, Germany

2 German Cancer Research Center (DKFZ) Heidelberg, Division of Redox Regulation, DKFZ-ZMBH Alliance, Germany

3 Faculty of Biosciences, Heidelberg University, Heidelberg, Germany

4 Institute of Pathology, Heidelberg University, Heidelberg, Germany

5 Center for Lung Research (DZL), Heidelberg, Germany

6 German Cancer Research Center (DKFZ) Heidelberg, Division of Applied Bioinformatics, Germany

7 Heidelberg Institute for Stem Cell Technology and Experimental Medicine, Heidelberg, Germany

8 Institute of Pharmacology, Medical University of Vienna, Vienna, Austria

9 Department of Pediatric Hematology, Oncology and Immunology, Heidelberg University, Heidelberg, Germany

10 German Cancer Research Center (DKFZ) Heidelberg, Research Group Cell Fate Engineering and Disease Modeling, Germany

11 HITBR Hector Institute for Translational Brain Research GmbH, Heidelberg, Germany

12 Central Institute of Mental Health, Medical Faculty Mannheim, Heidelberg University, Mannheim, Germany

13 Metabolomics Core Technology Platform, Centre for Organismal Studies (COS), Heidelberg University, Heidelberg, Germany

14 German Cancer Research Center (DKFZ) Heidelberg, Department of Translational Oncology, Germany

15 German Cancer Consortium (DKTK), partner site Munich, a partnership between DKFZ and Ludwig-Maximilians-University, Munich, Germany

16 Department of Medicine III, LMU University Hospital, LMU Munich, Germany
17 University of Cologne, Faculty of Medicine and University Hospital Cologne, Department of Translational Genomics, Cologne, Germany
18 Technical University of Munich, School of Medicine and Health, Institute of Clinical Chemistry and Pathobiochemistry, TUM University Hospital, Munich, Germany
19 German Cancer Consortium (DKTK), Heidelberg, Germany

*To whom correspondence should be addressed: n.gunkel@dkfz.de

$ Equal contribution

Table of Contents

Supplementary results 4
Supplementary methods 5
Figure S1 11
Figure S2 13
Figure S3 15
Figure S4 17
Figure S5 19
Figure S6 21
Figure S7 22
Figure S8 24
Figure S9 26
Figure S10 28
Figure S11 30
Figure S12 32
Figure S13 34
Figure S14 36
Figure S15 37
Supplementary Table S1: Chemical compounds 39
Supplementary Table S2: Primers 40
Supplementary Table S3: Primary and secondary antibodies 40
Supplementary Table S4: qPCR primers 41
Supplementary Table S5: LC gradient for metabolomics 41
References 42

Supplementary results

A possible mechanism for the failure to induce ROS buffer genes could be the constitutive nuclear activity of BACH1, a transcriptional repressor of a subset of NRF2 response genes[1,2]. An earlier report has demonstrated that, in HaCaT cells, CDDO-Me not only increases NRF2-levels but also causes nuclear export of BACH1, leading to a more efficient NRF2 response[3]. Although we couldn't formally show nuclear export, we detected a reduction of nuclear BACH1 upon CDDO-Me in SCLC cells (Fig. S10A, left panel). The combination of the BACH1 inhibitor hemin[4] and CDDO-Me resulted in an increased induction of HMOX1, which has been reported to be positively regulated by NRF2 and repressed by BACH1[5] (Fig. S10A, right panel), confirming that the NRF2/BACH1 regulon is functional in SCLC cells. However, CDDO-Me/hemine pre-treatment did not enable SCLC cells to develop significant resistance against DKFZ-682 (Fig. S10B), arguing that continual transcriptional repression by BACH1 is not responsible for the low-ACB status and high drug sensitivity of SCLC cells. We also tested the hypothesis that SCLC achieve low-ACB expression through the downregulation of MAFG, a positive acting binding partner of NRF2[6]. In Beas-2B, but not in HaCaT cells, knock down of MAFG prevented resistance induction by CDDO-Me, suggesting that ROS buffer capacity in these cells is dependent on genes regulated by NRF2/MAFG heterodimers (Fig. S11A). We noted that in SCLC cell lines the MAFG promoter is hyper-methylated in regions where ARE consensus sequences have been predicted (Fig. S11B). As MAFG itself is a target of NRF2, it is plausible that its epigenetic repression in SCLC prevents a feed forward loop of ROS response, which had previously been predicted[7]. We circumvented epigenetic silencing of MAFG by tet-inducible overexpression. Our own data and a previous report[8] showed that excessive overexpression of MAF proteins can lead to the repression of NRF2 response genes. We therefor used doxycycline concentrations which allowed a robust but low MAFG induction and found, unexpectedly, that overexpression of MAFG did not enable SCLC cells to develop drug resistance upon NRF2 induction (Fig. S11C, D). This suggests that MAFG is not a limiting factor for the defense against drug induced ROS stress in SCLC cells.

Supplementary methods

Generation of MAFG and FOSL1 overexpressing cells

Sequences of primers used for cloning are provided in **Supplementary Table S2**. The ORF of human *MAFG* or *FOSL1* was PCR amplified from plasmid carrying this transgene with primers containing SalI and AvrII restriction sites using Phusion High-Fidelity Polymerase (Thermo Fischer Scientific) and inserted into the lentiviral pLIX_403 vector. The sequence of construct was confirmed by sequencing (Microsynth).

Lentivirus was produced through transfection of lentiviral backbones containing transgene (pLIX-*MAFG* or pLIX-*FOSL1*) along with third-generation packaging plasmids into HEK293T cells according to the Trono laboratory protocol[9]. Fresh lentivirus from transfected HEK293T culture supernatant was used for viral transduction, specifically 300 µL of fresh lentivirus was added to 1 million of suspension cells (H82, H69 and HCC33). All experimental procedures for lentivirus production and transduction were performed in a biosafety level 2 laboratory. 24 h after transduction, lentivirus was removed from the media by centrifugation (300 *g*, 3 min) and cells were kept with 3 mL of fresh medium in 6-well plates for 48 h. Puromycin (0.5 µg/ mL) was added to cells for antibiotic selection for one week. Puromycin resistant cells were kept without puromycin to recover and used for further experiments. The cells armed with a tet-inducible MAFG or FOSL1 expression construct were treated with doxycycline (0.06 -1 µg/mL for 24 h) to increase MAFG or FOSL1 expression and the level of protein was validated by Western blotting.

Monitoring of TXNRD1 activity in cells with TRFS-Green

The probe TRFS-Green was synthetized according to Zhang et al.[10]. Cells (7,500 cells/well) were seeded in V-bottom 96-well plates in FluoroBrite DMEM medium (Gibco) and incubated at 37 °C in 5 % CO_2 atmosphere. After four hours, DKFZ-608 was added at indicated concentrations in triplicates, and TRSF-Green (10 µM) was immediately added. Following mixing with a multi-channel pipette, plates were centrifuged at 200 *g* for 5 min. Fluorescence (450/520 nm) was monitored using a CLARIOstar plate reader at 2 min intervals over 8 h at 37 °C. Dose-response curves were generated by calculating the fluorescence increase during the linear phase (e.g. 100 min) for each well. Mean values +/- SD were plotted.

To investigate the effect of CDDO-Me pre-treatment on TXNRD1 activity, cells were seeded in a 12-well plate (100,000 cells per well for HaCaT and Beas-2B, 500,000 cells per well for H69, H82, and H526). The next day, the pre-treatment with 50 nM CDDO-Me was started for 24 h. Afterwards, cells were harvested and washed with PBS (centrifuged for 3 min at 300 g). Cell pellets were resuspended in 200 µL of PBS (400 µL for HaCaT). For the TXNRD1 activity assay on a 96-well plate, 5 µL of cell suspension was mixed with 100 µL of the FluoroBrite DMEM medium supplemented with 2 % FCS containing different dilutions of DKFZ-682 (0, 0.2, or 20 µM). Finally, 10 µM TRFS-Green was added to each well and fluorescence was recorded with the CLARIOstar plate reader in 2 min intervals for 2 hours at 37 °C using the filters 438/15 for excitation and 538/20 for emission. Protein concentrations in cell suspensions were determined by the Pierce BCA assay using BSA as a standard. Each sample and condition were measured as a technical triplicate in each experiment and the experiment was repeated three times independently. The slope of Δ fluorescence per s was calculated in the linear phase, which was the time range of 40–100 min. TXNRD1 activity was expressed as Δ fluorescence per s per mg of protein.

Gel electrophoresis and Western blotting

For total protein extraction, cells were harvested using Pierce™ RIPA buffer (Thermo Scientific, 89900) supplemented with a protease inhibitor cocktail (Serva), 100 U/ml benzonase (Merck) and, if necessary, PhosSTOP phosphatase inhibitor cocktail (Roche). Protein concentrations were measured by Pierce BCA Protein assay using BSA as a standard.

For preparation of cytoplasmic and nuclear extracts, the cells were washed twice with cold PBS and resuspended in Hypotonic Buffer (10 mM HEPES (pH 7.9), 0.1 mM EDTA, 10 mM KCl, 1 mM dithiothreitol (DTT), 0.7 % Nonidet P-40, and protease inhibitor mixture). After incubation on ice for 10 min, the nuclei were pelleted by centrifugation (13 000 rpm at 4 °C, 30 s), and the supernatant (cytoplasmic fraction) was transfered to a clean microcentrifuge tube and stored at -20 °C. The pellet (nulear fraction) was washed with Hypotonic Buffer, and finally resuspended in lysis buffer (50 mM HEPES (pH 7.9), 10 % glycerol, 0.3 M NaCl, 50 mM KCl, 0.1 mM EDTA, 1 mM DTT, and protease inhibitors). After incubation on ice for 20 min, the nuclear extracts were centrifuged at 13,000 rpm at 4 °C for 10 min, and the supernatant (nuclear fraction) was stored at -20 °C.

Flow cytometry

As an indicator of ferroptosis, lipid peroxidation was analyzed in cells stained with Bodipy 581/591 C11 (Invitrogen). Cells were stained with 3 µM Bodipy 581/591 C11 diluted in the culture medium for 30 min at 37 °C. Afterwards, cells were washed with PBS, and analyzed using the flow cytometer BD LSR Fortessa (BD Biosciences). The ratio of the oxidized (excitation 488 nm, emission 530/30) and reduced (excitation 561 nm, emission 610/20) dye was calculated for each cell in the FlowJo software.

For detection of ROS/RNS level 150,000 cells/well/1 mL (adherent cells) or 500,000 cells/mL (suspension cells) were seeded in 12-well plate. The next day, cells were treated with DMSO (unstained control), 5 µM CM-H2DCFDA (Invitrogen, Thermo Scientific, Cat No. C6827), 1:250 OxiVision Green peroxide sensor (AAT Bioquest, Cat No. 11506, powder was solved in 200 µL DMSO) or 5 µM DAF-FM Diacetate (Invitrogen, Thermo Scientific, Cat No. D23844) at 37 °C for 30 min; 1:400 DAX-J2™ PON Green (AAT Bioquest, Cat No. 16317) at 37 °C for 60 min. Then cells were detached with trypsin (adherent cells) or washed (suspension cells), spun down 5 min at 1200 rpm and finally each cell pellet was resuspended in 500 µL PBS with 1 % FBS and analyzed by the flow cytometer Guava easyCyte 14HT (Luminex). The fluorescence of all above-mentioned dyes was analyzed in the Green-B channel (excitation 488 nm, emission 512/18 nm) while counting 10,000 cells per sample. For ROS Brite 570 (10 µM, AAT Bioquest, Cat No. 16000) staining, cells were first detached with trypsin (adherent cells) or washed (suspension cells) and then incubated with ROS Brite 570 at 37 °C. After 20 min cells were spun down 5 min at 130 g and cell pellet was resuspended in 500 µL PBS with 1 % FCS and analyzed by flow cytometry (Yellow-G channel: excitation 532 nm, emission 575/25).

Treatment: cells were co-incubated with OxiVision Green peroxide sensor and drug; pre-incubated with drug, then detached with trypsin (adherent cells) or spun down (suspension cells), washed and further incubated with ROS Brite 570. Stained but untreated cells were used as control.

Measurement of ROS levels in cytoplasm and mitochondria

The effect of drugs on roGFP2-Orp1 oxidation was quantified as described[11] in the NSCLC cell line H838 stably expressing roGFP2-Orp1 (with or without the mitochondrial targeting sequence). The day before the measurement cells were seeded into a black clear-bottomed 96-well imaging plate (Falcon, Cat No. 353219) at a density of 20,000 cells/well in 200 µL Fluorobrite medium. A non-transduced control was included on the same plate for background subtraction. In order to obtain the fluorescence intensity values

for a fully oxidized and reduced probe, control wells were treated with 2 mM diamide or 10 mM DTT for 15 min at 37 °C. After the entire plate was measured for 8 cycles in a CLARIOstar fluorescence plate reader, BMG Labtech (which allows the simultaneous detection of the two excitation maxima of roGFP2 (400 nm and 485 nm) when emission is monitored at 520 nm), 22 µL of 10x concentrated drug was added and measurement continued for up to 340 min. The readout of the roGFP2 measurement was expressed as the degree of sensor oxidation (OxD, see equation in reference 15 . All treatments were performed in technical triplicate seeded in different quadrants of the imaging plate, to avoid position effects.

Lactate Dehydrogenase (LDH) Release Assay

Lactate dehydrogenase (LDH) release was measured by using the LDH-GloTM Cytotoxicity Assay (Promega, Cat No. J2380), according to the manufacturer's instructions. Briefly, H1105 and H82 cell lines were seeded in 96-well plates at a density of 20,000 cells/well in 100 µL of culture medium with 10 % serum. After 24 h, test compound was added (100 µL per well, all concentrations in triplicate) without medium change. Following treatment, supernatants were collected and diluted 1:20 into LDH Storage Buffer (200 mM Tris-HCl (pH 7.3), 10 % Glycerol, 1 % BSA) and sore at -20 °C for the assay. LDH activity was measured by adding an equal volume of LDH Detection Reagent to the diluted sample and incubated for 40 min at room temperature, protected from light. The luminescence was recorded using a microplate reader. Maximum LDH release was determined by lysing control cells with the lysis solution (10% Triton X-100). Wells without cells served as a negative control to determine culture medium background.

Metabolomic analysis of nucleotide intermediates

To analyze nucleotide intermediates (NADP+ and NADPH), adherent cells (HaCaT, Beas-2B) were quickly rinsed with ice-cold saline (0.9 % NaCl) and scraped off into 1 mL of ice-cold saline. Cell suspension was transferred into a microtube and spun down at 500 g for 1 min at 4 °C. After removal of supernatant, cell pellets were snap-frozen in liquid nitrogen and stored at -80 °C until further processing. Suspension cells were first pelleted at 500 g for 2 min at 4 °C in 15-mL tubes. Pellets were resuspended in 1 mL of ice-cold saline, cell suspension was transferred into a microtube and spun down at 500 g for 2 min at 4 °C. After removal of supernatant, cell pellets were snap-frozen in liquid nitrogen and stored at -80 °C until further processing. Frozen cell pellets (1-5 mio cells) were processed following an adjusted protocol targeting energy carriers such as NAD/NADH and NADP/NADPH[12]. This method was extended by including polarity

switching and additional metabolites of interest. Briefly, on ice to the frozen cell pellets were added ice-cooled steel balls with 250 µl cooled extraction buffer (Acetonitrile: Methanol: 15 mM ammonium acetate in H2O (3:1:1), pH 10) and 2.5µL of 10µM d5-Tryptophan in water. Subsequently, samples were sonicated for 5min on ice and cortex vigorously. Afterwards, samples were centrifuged for 15 min at 4 °C and 13,000g, and the resulting supernatant was transferred to a new LC-MS grade autosampler vial for measuring.

For metabolite separation and detection, an ACQUITY I-class PLUS UPLC system (Waters) coupled to a QTRAP 6500+ (AB SCIEX) mass spectrometer with electrospray ionization (ESI) source was used. In detail, metabolites were separated on an ACQUITY Premier BEH Amide Vanguard Fit column (100 mm × 2.1 mm, 1.7 µm, Waters) with constant column temperature of 35 °C. Separation of NAD/NADH, NADP/NADPH and additional energy carriers was achieved by the following LC gradient scheme (Supplementary Table S5) using mobile phase A (50/50; Acetonitrile / Water with 5 mM ammonium acetate + 0.05% (v/v) ammonium hydroxide, pH 10) and mobile phase B (90/10; Acetonitrile : Water with 5 mM ammonium acetate + 0.05% (v/v) ammonium hydroxide, pH 10). Data acquisition was performed using Analyst 1.7.2 (AB SCIEX) and processed using the OS software suite 2.0.0 (AB SCIEX). After extraction, pelleted cell debris was lysed in PierceTM RIPA buffer (Thermo Scientific, 89900) supplemented with a protease inhibitor cocktail (Serva) and sonicated on ice for 10 s using a probe sonicator (10 % amplitude). The protein concentration in lysates was measured using Pierce BCA Protein Assay Kit (Thermo Scientific) with BSA as a standard. Metabolite results were expressed as unit less peak area corrected with d5-tryp as an internal standard for batch effect correction. The data were normalized to protein amount.

Detection of glutathione

Intracellular GSH levels were quantified using the Reduced Glutathione Assay Kit (Abcam, ab235670) following the manufacturer's instructions. To prepare the samples, adherent cells (HaCaT, Beas-2B) were detached using trypsin, washed first with culture medium to neutralize trypsin activity, and then washed with PBS. One million pelleted cells were resuspended in 30 µL of 5 % sulfosalicylic acid (SSA) solution and incubated on ice for 10 min. After centrifugation at 12,000 g at 4°C for 10 min, the supernatant was collected as the sample solution. The samples were then diluted 20-fold with GSH Assay Buffer, and 10 µL of the diluted samples were added to the wells of a black 96-well plate. The sample volumes were adjusted to 20 µL with GSH Assay Buffer, followed by the addition of 80 µL of Reaction Mix to each well.

Fluorescence was measured in kinetic mode at room temperature for 60 min with 2-minute intervals in a CLARIOstar plate reader (BMG LABTECH).

Intracellular levels of glutathione (GSH and GSSG) in the cells were determined using the GSH/GSSG-GloTM Assay (Promega, V6611). Cells (HaCaT and Beas-2B: 2 × 10^5 cells in 2 mL of medium per well in a 6-well plate; H82 and H69: 10^6 cells in 2 mL of medium per well in a 12-well plate) were seeded. The following day, the test compound was added to each well and incubated for 24 h. Then, the cells were harvested with lysis buffer (0.5% NP-40 in PBS) with or without N-ethylmaleimide (NEM, 2.5 mM), and glutathione levels were quantified using the GSH/GSSG-Glo™ Assay according to the manufacturer's instructions. Luminescence was measured using a FLUOstar OPTIMA plate reader (BMG LABTECH).

Hoechst 33342/propidium iodide double staining

The cells seeded in a 96-well plate were incubated with Hoechst 33342 (1 µg/mL) and propidium iodide (1 µg/mL) in a dark incubator at 37 °C for 30 min. Images were obtained using a Leica fluorescence microscope (40x objective magnification) combined with a digital camera and Las Ez (version 3.1.0) software.

Immunofluorescence

Twelve-well plates were coated with poly-L-lysine (Gibco, A38904) for 1 h at room temperature (RT) and washed three times with sterile H_2O. Cells were seeded at a density of 0.5 × 10^6 cells in 1 ml per well. On the following day, cells were treated with doxycycline. Next day, cells were fixed with 4% paraformaldehyde in PBS for 10–20 min at RT and washed three times with PBS. Cells were permeabilized with 0.2% Triton X-100 in PBS for 10 min and washed three times with PBS. Non-specific binding was blocked using 1% bovine serum albumin (BSA) in PBS for 30–60 min at RT. Samples were incubated overnight at 4 °C with anti-Fra-1 (Abcam, ab252421) primary antibody diluted 1:500 in 1% BSA/PBS. After three washes with PBS (5 min each), cells were incubated for 45 min in the dark at RT with goat anti-rabbit Alexa Fluor 594 secondary antibody (ThermoFischer, A-11037) diluted 1:1000 in 1% BSA/PBS together with Hoechst 33342 (1 µg/mL). Cells were washed three times with PBS (5 min each). Images were obtained using a Leica fluorescence microscope combined with a digital camera and Las Ez (version 3.1.0) software.

Figure S1

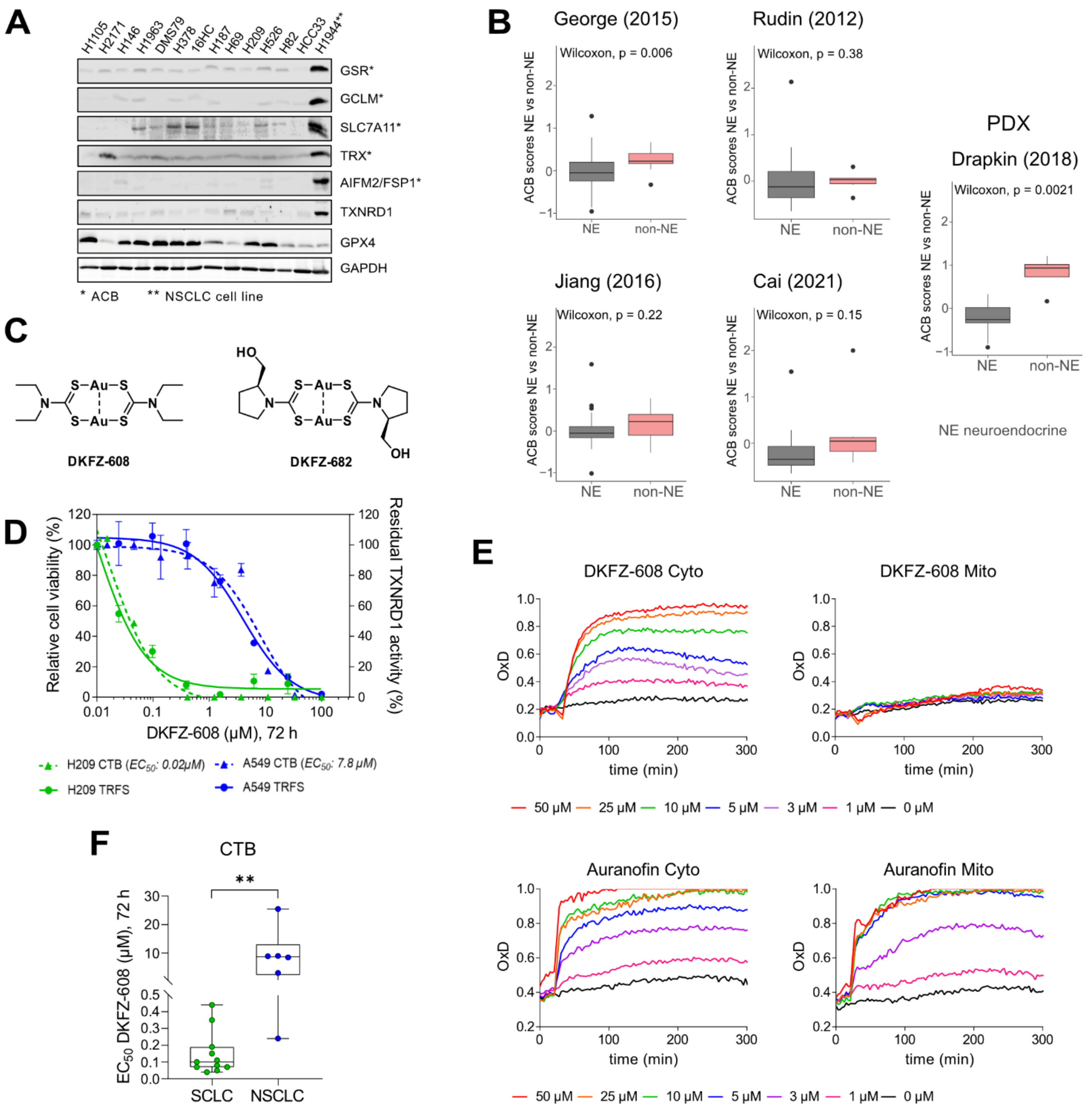

Figure S1: SCLCs express low levels of ACB proteins independent of the neuroendocrine subtype. (A) Protein levels of selected ACBs and two additional redox proteins, TXNRD1 and GPX4, in total cell extracts

from 13 SCLC cell lines were analyzed by immunoblotting (*n* = 2). High ACB protein expression in drug resistant H1944 NSCLC cell line was used as a reference. **(B)** The ACB status of SCLC cells derived from PDX models and patient samples showing neuroendocrine (NE) and non-NE characteristics. Expression data were downloaded from the Dryad Digital Repository (https://datadryad.org/stash/share/BkmPd Mrwhae1VxhkkSLIG_532FLCqcYiMFUpY1yKmGA)[13]. SCLC samples were categorized into four expression-based subtypes (ASCL1, NEUROD1, POU2F3 and YAP1) based on the highest expression level of key transcription factors. These subtypes were further grouped into "neuroendocrine" (ASCL1 and NEUROD1) and "non-neuroendocrine" (POU2F3 and YAP1) categories to enable comparative analyses between these biologically distinct groups. ACB scores were calculated by taking the average of the scaled expression values for the ACB-genes. Box plots show the median and the interquartile range, and whiskers extend to 1.5x the interquartile range. Significance was determined using the Wilcoxon test.

An impact of DKFZ-608 (TXNRD1 inhibitor) on cell viability and cellular redox status. (C) Chemical structure of DKFZ-608 and DKFZ-682, previously reported as gold (I)-dithiocarbamate (dtc) complex 5 and complex 37 respectively[14]. **(D, F)** SCLC and NSCLC cell lines were treated with a range of concentrations of DKFZ-608 for 72 h. Cell viability was assessed using CellTiter-Blue (CTB) assay (**$p < 0.01$, two-tailed unpaired *t*-test). **(D)** To quantify intracellular TXNRD1 inhibition, cell lines were treated with TRFS, immediately followed by a dilution series of DKFZ-608. Fluorescence induction was measured for 8 h and dose response effects were analyzed at time point 100 min. All data were normalized (untreated control was set to 100 %). Data are presented as mean ± SD of three technical replicates. **(E)** H838 cells expressing either cytoplasmic (Cyto) or mitochondrial (Mito) roGFP2-Orp1 were treated with various concentrations of DKFZ-608 or auranofin. Cells were treated with sulphobuthylester-ß-cyclodextrin as a solvent control for DKFZ-608 and with DMSO as a control for auranofin (black lines). The fluorescence signals from oxidized and non-oxidized cytoplasmic or mitochondrial roGFP2-Orp1 were monitored for 300 min. The oxidation degree (OxD) represents the extent of probe oxidation, with data normalized to 1.0 (fully oxidized) defined by the signal from diamide (2 mM) and 0.0 (fully reduced) defined by the signal from DTT (10 mM). Results are representative of two independent experiments each performed in triplicate. Source data are provided as a Source Data file.

Figure S2

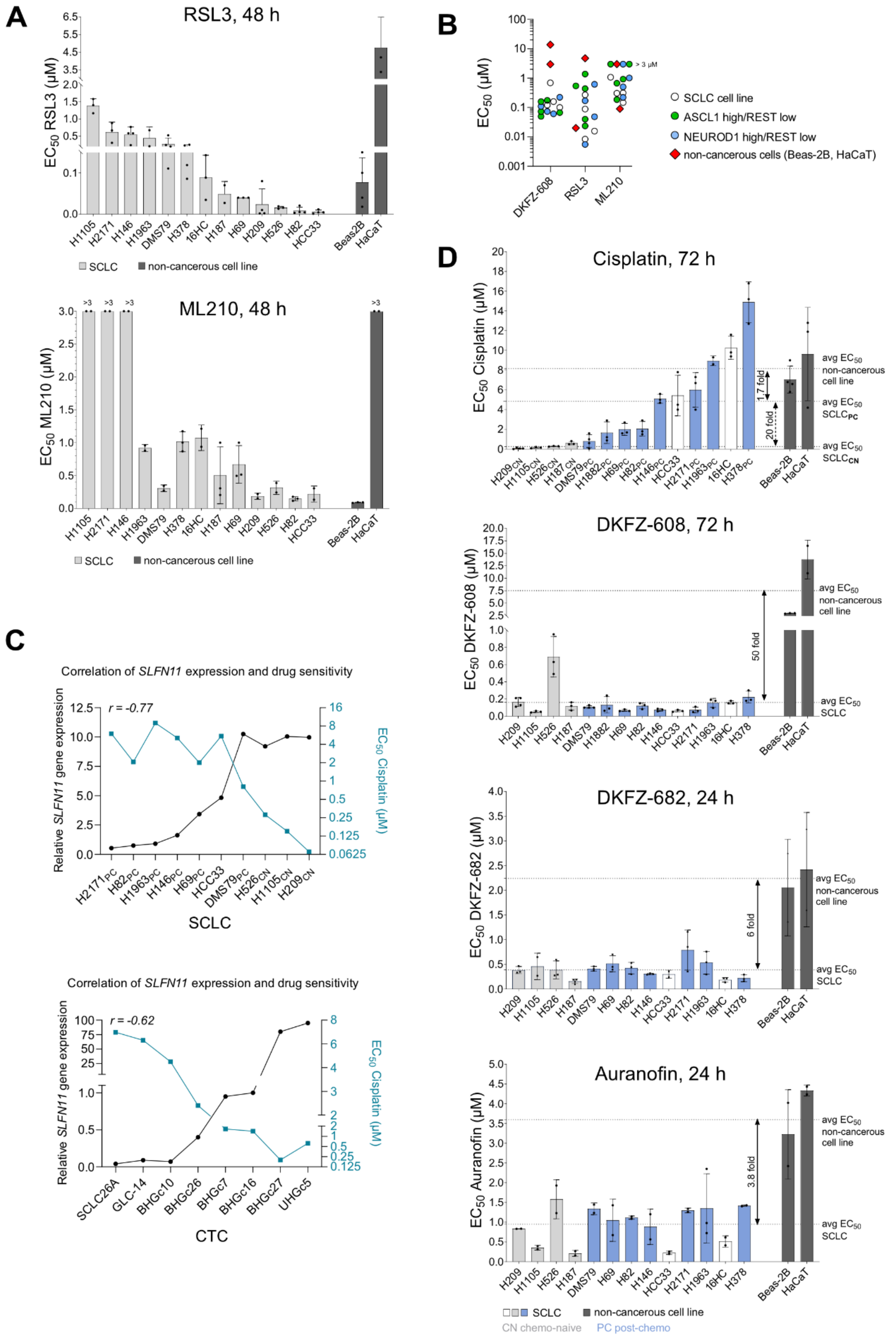
A
RSL3, 48 h
EC50 RSL3 (µM)
H1105 H2171 H146 H1963 DMS79 H378 16HC H187 H69 H209 H526 H82 HCC33 Beas2B HaCaT
SCLC
non-cancerous cell line
ML210, 48 h
EC50 ML210 (µM)
>3
B
EC50 (µM)
> 3 µM
DKFZ-608
RSL3
ML210
SCLC cell line
ASCL1 high/REST low
NEUROD1 high/REST low
non-cancerous cells (Beas-2B, HaCaT)
C
Correlation of SLFN11 expression and drug sensitivity
r = -0.77
Relative SLFN11 gene expression
EC50 Cisplatin (µM)
H2171PC H82PC H1963PC H146PC H69PC HCC33 DMS79PC H526CN H1105CN H209CN
SCLC
r = -0.62
SCLC26A GLC-14 BHGc10 BHGc26 BHGc7 BHGc16 BHGc27 UHGc5
CTC
D
Cisplatin, 72 h
EC50 Cisplatin (µM)
avg EC50 non-cancerous cell line
avg EC50 SCLCPC
avg EC50 SCLCCN
1.7 fold
20 fold
DKFZ-608, 72 h
EC50 DKFZ-608 (µM)
avg EC50 SCLC
50 fold
DKFZ-682, 24 h
EC50 DKFZ-682 (µM)
6 fold
Auranofin, 24 h
EC50 Auranofin (µM)
3.8 fold
SCLC
non-cancerous cell line
CN chemo-naive
PC post-chemo

Figure S2: The cytotoxic effect of TXNRD1 inhibition is equal high for all tested SCLC cell lines, independent of their resistance to the GPX4 inhibitors 1S3R-RSL3 (RSL3) and ML210 or to cisplatin. (A-D) SCLC and non-cancerous cell lines were treated with different concentration of drugs for the indicated time point and the cell viability was quantified by the CellTiter-Glo assay. Cells were treated with DMSO as a control for RSL3, ML210, cisplatin and auranofin, and with sulphobuthylester-ß-cyclodextrin as a solvent control for DKFZ-608 and DKFZ-682. Bar diagrams show the mean ± SD of EC_{50} data from at least two independent experiments, each performed in biological triplicate. **(B)** NE scores were derived from the Gazdar Small Cell Lung Cancer Neuroendocrine Explorer[15]**. (C)** The data for SLFN11 gene expression in SCLC cell lines and circulating tumor cells (CTC) are from DepMap and Gerhard Hamilton, respectively (*r*, Pearson correlation). Source data are provided as a Source Data file.

Figure S3

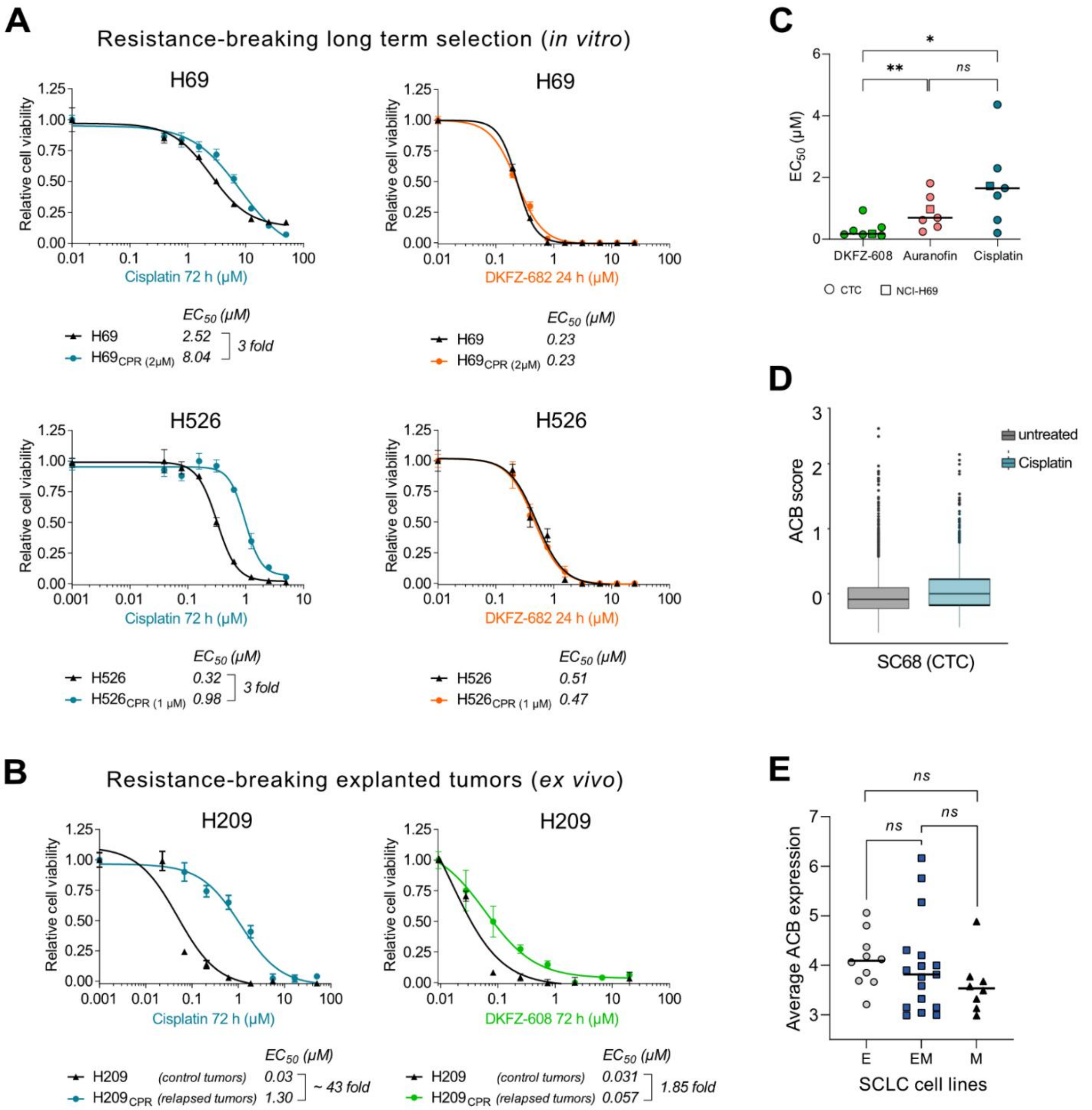

Figure S3: Cisplatin-resistant cells remain sensitive to TXNRD1 inhibition. (A) Parental H69 and H526 and cisplatin-resistant (CPR) H69 (resistant to 2 µM of cisplatin) and H526 (resistant to 1 µM of cisplatin) cell lines were exposed to cisplatin for 72 h or DKFZ-682 for 24 h. The cell viability was quantified by the CellTiter-Glo assay. Data represents the mean ± SD of three biological replicates from one of two independent experiments. **(B)** H209 cells, isolated from an etoposide/cisplatin-resistant tumor after 3

cycles *in vivo* therapy, and naïve H209, isolated from control tumors, were treated with various concentrations of cisplatin or DKFZ-608 for 72 h. Surviving cells were quantified by CellTiter-Blue assay. Data are presented as mean ± SD of three biological replicates. **(C)** SCLC H69 cell line and circulating tumor cells (CTC) were seeded in 96 well plates as two-dimensional cultures. EC_{50} values were determined from dose response curves after 96 h treatment with the indicated drugs using a modified MTT assay. Each dot represents a mean of three technical replicates (ns, not significant, *$p < 0.05$, **$p < 0.01$, ratio paired *t*-test).

Circulating tumor cells (CTC) maintain low ACB expression after cisplatin therapy. (D) CTCs from naïve and relapsed patients. Data from Stewart et al. 2020 were obtained from the GEO database (accession number GSE138474) and processed by sub-setting and normalizing data from SC68 to focus on epithelial cells. Log counts values were scaled, and the expression levels of ACB genes were averaged to calculate the ACB score. Vehicle-treated and cisplatin-treated cells are compared to assess the impact of treatment. Box plots were used to display the data, with the median and interquartile range shown, and whiskers extending to 1.5 times the interquartile range. Statistical significance was evaluated using the Wilcoxon test.

SCLC cells with distinct profiles of EMT markers demonstrate non-significant differences in ACB expression. (E) 34 SCLC cell lines were separated according to pre-calculated EMT signatures[16] into epithelial (E), mesenchymal (M) and mixed groups (EM). Based on paired *t*-tests, differences of ACB expression (average values of log2 (TPM+1) converted reads) between groups are non-significant (ns).

Source data are provided as a Source Data file.

Figure S4

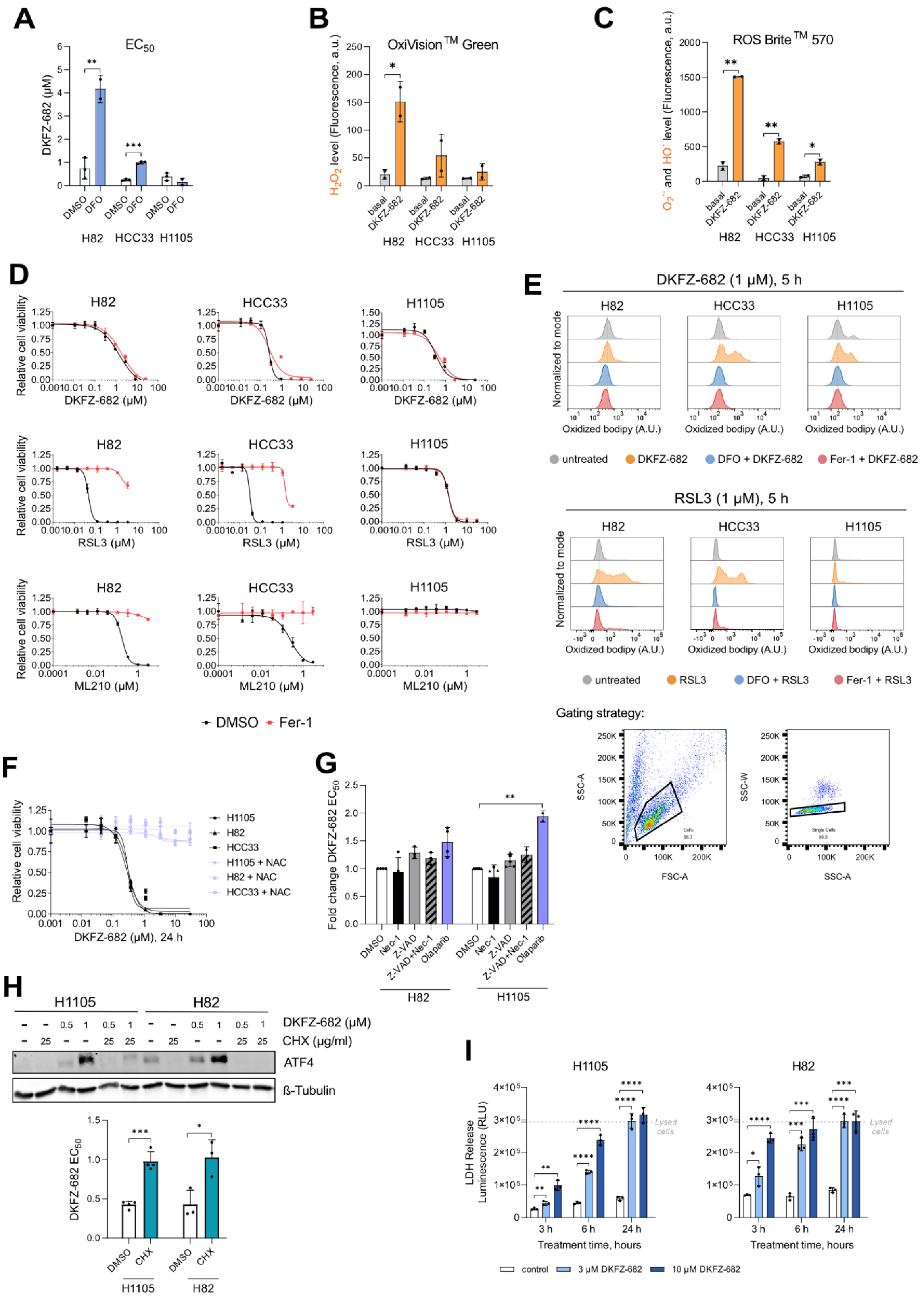
A
EC50
DKFZ-682 (µM)
DMSO
DFO
H82
HCC33
H1105
B
OxiVision™ Green
H_2O_2 level (Fluorescence, a.u.)
basal
DKFZ-682
C
ROS Brite™ 570
$O_2^{\cdot-}$ and $HO^{\cdot}$ level (Fluorescence, a.u.)
D
Relative cell viability
DKFZ-682 (µM)
RSL3 (µM)
ML210 (µM)
DMSO
Fer-1
E
DKFZ-682 (1 µM), 5 h
Normalized to mode
Oxidized bodipy (A.U.)
untreated
DFO + DKFZ-682
Fer-1 + DKFZ-682
RSL3 (1 µM), 5 h
RSL3
DFO + RSL3
Fer-1 + RSL3
Gating strategy:
SSC-A
FSC-A
SSC-W
Cells
Single Cells
F
DKFZ-682 (µM), 24 h
H1105
H82
HCC33
H1105 + NAC
H82 + NAC
HCC33 + NAC
G
Fold change DKFZ-682 EC50
DMSO
Nec-1
Z-VAD
Z-VAD+Nec-1
Olaparib
H
DKFZ-682 (µM)
CHX (µg/ml)
ATF4
ß-Tubulin
DKFZ-682 EC50
CHX
I
LDH Release
Luminescence (RLU)
Lysed cells
Treatment time, hours
3 h
6 h
24 h
control
3 µM DKFZ-682
10 µM DKFZ-682

Figure S4: Cell death mechanisms induced by TXNRD1 inhibition in SCLC. (A) Cells were pre-treated with DMSO or DFO (100 µM) for 1 h and then treated with a concentration series of DKFZ-682 for 24 h and cell viability was measured by the CellTiter-Glo assay (EC_{50} mean ± SD, at least of two independent experiments, each performed in three biological replicates; $**p < 0.01$, $***p < 0.001$, two-tailed unpaired *t*-test). **(B, C)** Cells were stained with the OxiVision™ Green peroxide sensor or ROS Brite™ 570 fluorescent dye upon treatment with DKFZ-682 (20 µM, 30 min) and analyzed by flow cytometry. Data are presented as mean ± SD of two independent experiment ($*p < 0.05$, $**p < 0.01$, two-tailed unpaired *t*-test). **(D)** Cell viability of indicated cell lines, pre-treated with ferrostatin-1 (Fer-1, 5 µM) for 1 h and then treated with DKFZ-682 (24 h), RSL3 or ML210 (48 h), was analyzed by CellTiter-Glo assay. The graphs are representative of two independent experiments each performed in biological triplicate. **(E)** Cells were pre-treatment with DFO (100 µM) or Fer-1 (5 µM) for 1 h and then treated with DKFZ-682 or RSL3 for 5 h. To analyze lipid peroxidation, cells were stained with bodipy 581/591 C11 and analyzed by flow cytometry. The displayed graphs are representative of 2 independent experiments. At the bottom, the gating strategy is shown. **(F)** HCC33, H1105 and H82 cell lines were treated with or without 3 mM N-acetylcysteine (NAC) for 30 min, followed by a 24-hour treatment with a concentration series of DKFZ-682. Cell viability was measured by the CellTiter-Glo assay. The graph is representative of two independent experiments each performed in biological triplicate. **(G)** H1105 and H82 cell lines were pre-treated with DMSO, necrostatin-1 (Nec-1, 10 µM), Z-VAD-FMK (20 µM) alone or in combination (Z-VAD + Nec-1), or olaparib (100 nM). Then the cells were treated with a concentration series of DKFZ-682 for 24 h and the cell viability was measured by the CellTiter-Glo assay. Results are shown as the fold change in EC_{50} for DKFZ-682 in the presence of cell death inhibitors compared to the DMSO-treated control (at least of two independent experiments, each performed in three biological replicates; $*p < 0.05$, two-tailed unpaired *t*-test). **(H)** Cells were pre-treated with DMSO (control) or cycloheximide (CHX, 25 µg/ml) for 1 h and then treated with DKFZ-682 for 24 h. Protein levels of ATF4 in total lysate were analyzed by Western blotting (representative of 2 independent experiments). Cell viability was analyzed by CellTiter-Glo assay. The graph (EC_{50} mean ± SD; $*p < 0.05$, $***p < 0.001$, two-tailed unpaired *t*-test) summarizes the data of two to three independent experiments each performed in biological triplicate. **(I)** Cells were treated with the lowest full effect dose (3 µM) or 10 µM of DKFZ-682, and lactate dehydrogenase (LDH) release was quantified at 3, 6, and 24 hours post-treatment. The LDH level in lysed cells served as a reference for maximum release. Three biological replicates were performed for each cell line. Significance of differences was calculated with a two-tailed unpaired t-test ($*p < 0.05$, $**p < 0.01$, $***p < 0.001$, $****\ p < 0.0001$). Source data are provided as a Source Data file.

Figure S5

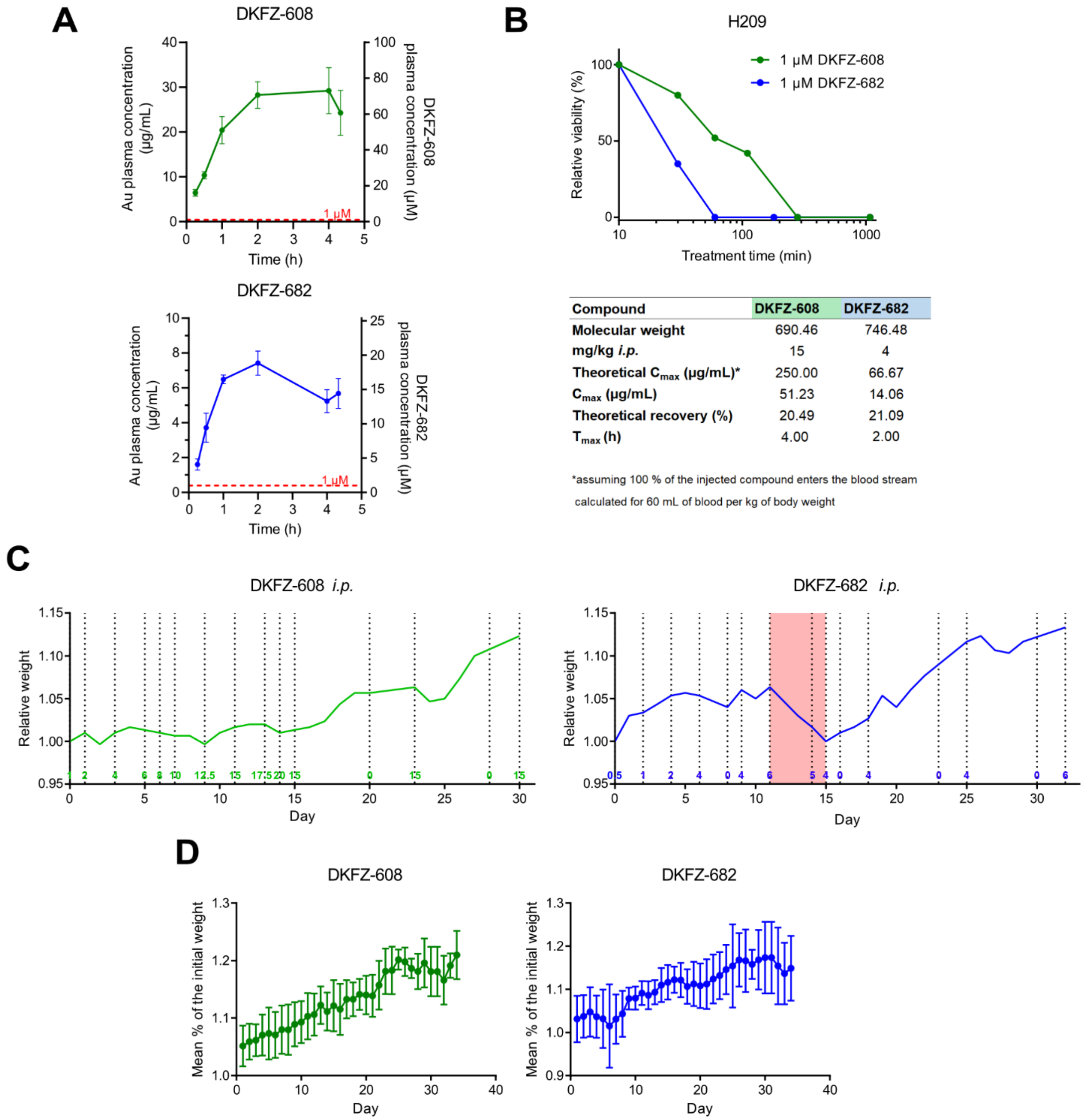

Compound	DKFZ-608	DKFZ-682
Molecular weight	690.46	746.48
mg/kg *i.p.*	15	4
Theoretical C_{max} (µg/mL)*	250.00	66.67
C_{max} (µg/mL)	51.23	14.06
Theoretical recovery (%)	20.49	21.09
T_{max} (h)	4.00	2.00

*assuming 100 % of the injected compound enters the blood stream
calculated for 60 mL of blood per kg of body weight

Figure S5: (A) Quantification of plasma levels of gold after i.p. application (left y-axis) and calculated drug concentration (right y-axis). Drugs were applied at the MTD of each compound (DKFZ-608 15 mg/kg, DKFZ-682 4 mg/kg). Dashed line indicates 1 µM drug levels at which DKFZ-608 requires 300 min and DKFZ-682 requires 60 min to eradicate H209 spheroids. **(B)** Quantification of cell killing in a time course using 1 µM

DKFZ-608 or DKFZ-682. Solid red and green line: H209 cells were cultured at high density (200,000 cells per well in V-bottom 96 well plates) and incubated with 1 µM DKFZ-608 or DKFZ-682. At the indicated time, drug containing medium was discarded, cells were washed and incubated with drug free medium up to time point 1080 min (18 h). Viability (metabolic activity, assessed by CTB) is calculated relative to control cells which have undergone the same procedure with drug-free medium. **(C)** Effect of dose escalation of DKFZ-608 and -682 on the weight gain of i.p. injected mice. The MTD of DKFZ-608 was defined as 15 mg/kg and of DKFZ-682 as 4 mg/kg. **(D)** Effect of 34 consecutive days of treatment with DKFZ-608 and 682 on the body weight of mice (For details see method section). Source data are provided as a Source Data file.

Figure S6

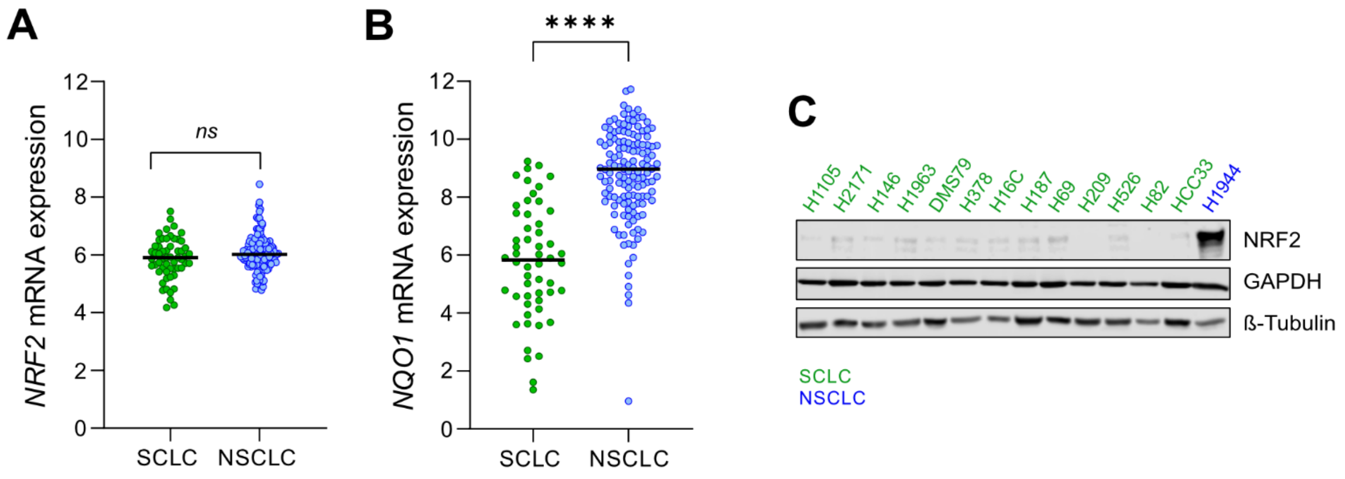

Figure S6: NRF2 transcript levels do not differ between SCLC and NSCLC. (A) NRF2 gene expression (data from DepMap) in cells grouped according to their origin (SCLC, NSCLC) does not differ significantly between SCLC and NSCLC (*ns*, not significant, two-tailed unpaired *t*-test).

NQO1*, a cell type independent proxy for NRF2 activity is expressed at lower levels in SCLC. (B)** *NQO1* gene expression data are from DepMap (* $p < 0.0001$, two-tailed unpaired *t*-test).

SCLC cell lines express lower levels of NRF2 protein, compared to the KEAP1 mutant cell line H1944. (C) Representative immune blot ($n = 2$) showing the protein level of NRF2 in total cell extracts from 13 SCLC cell lines. NRF2 expression in drug resistant H1944 NSCLC cell line was used as a reference.

Source data are provided as a Source Data file.

Figure S7

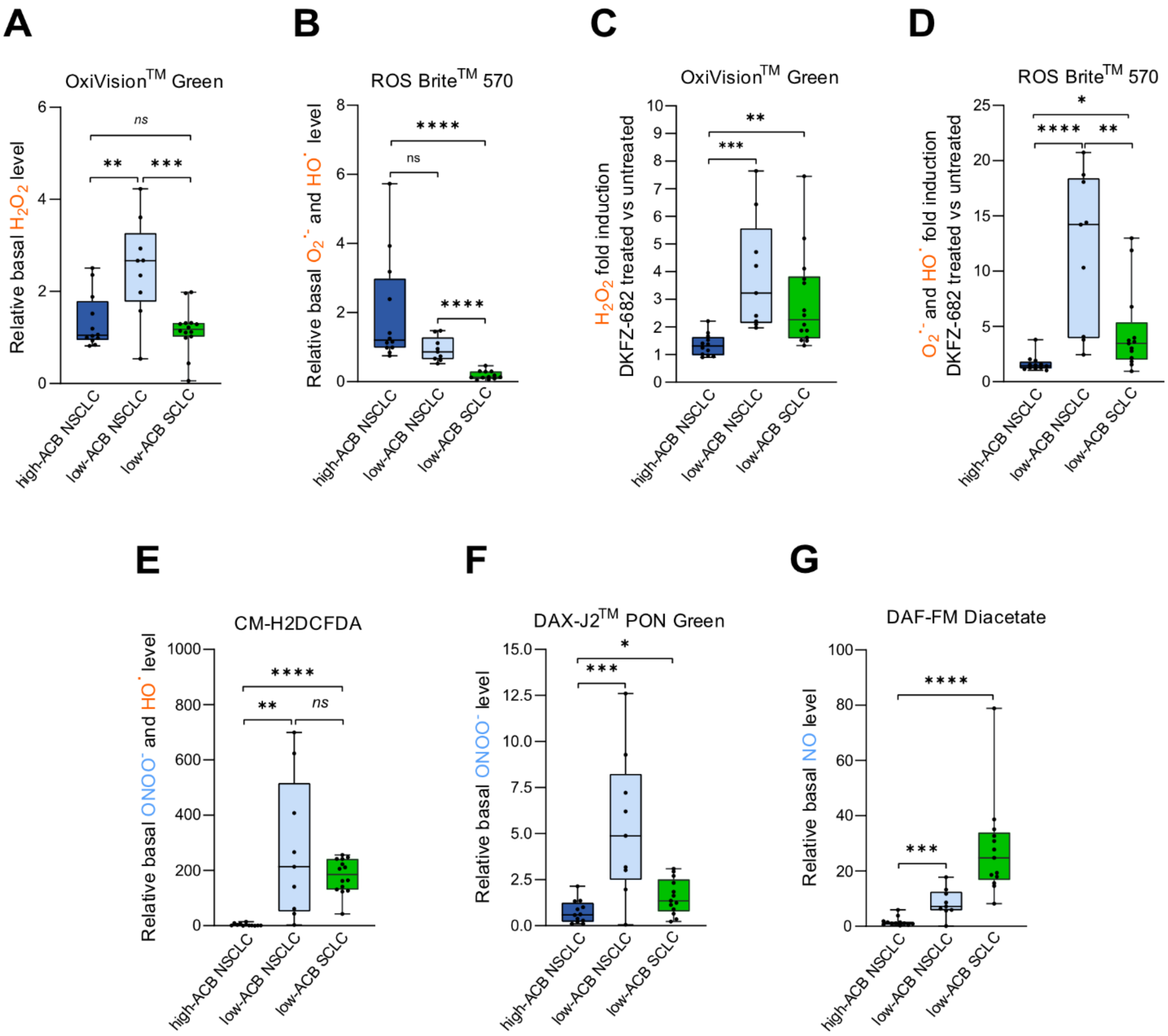

Figure S7: Basal and drug induced ROS and RNS levels in SCLC cell lines. A simplified schematic representation of the main reactive oxygen and nitrogen species (ROS – orange; RNS – blue) and sensors for their detection was described in our previous work[17]. Sensors are nonfluorescent cell-permeant reagents and produce bright fluorescence upon ROS/RNS oxidation. The reagents have good selectivity to H_2O_2 (OxiVision™ Green peroxide sensor), $O_2{\cdot}^-$ (ROS Brite™ 570), HO· (ROS Brite™ 570, CM-H2DCFDA), $ONOO^-$ (CM-H2DCFDA, DAX-J2™ PON Green) and NO (DAF-FM Diacetate). The basal ROS **(A, B)** and RNS **(E-G)** levels in high-ACB NSCLC cell lines (*n* = 12), low-ACB NSCLC cell lines (*n* = 9) and low-ACB SCLC cell lines (*n* = 13) were measured by staining with the OxiVision™ Green peroxide sensor **(A)**, ROS Brite™ 570

fluorescent dye[18] **(B)**, CM-H2DCFDA **(E)**, DAX-J2TM PON Green[19] **(F)**, and DAF-FM Diacetate[20] **(G)** and analyzed by flow cytometry. To investigate the changes in ROS levels upon treatment with DKFZ-682 (20 µM for 30 min), cell lines were stained with OxiVisionTM Green peroxide sensor **(C)** and ROS BriteTM 570 **(D)**, analyzed by flow cytometry and fold changes were calculated. Each dot (cell line) represents a mean of at least two independent experiments (*$p < 0.05$, **$p < 0.01$, ***$p < 0.001$, ****$p < 0.0001$, two-tailed unpaired *t*-test). Source data are provided as a Source Data file.

Figure S8

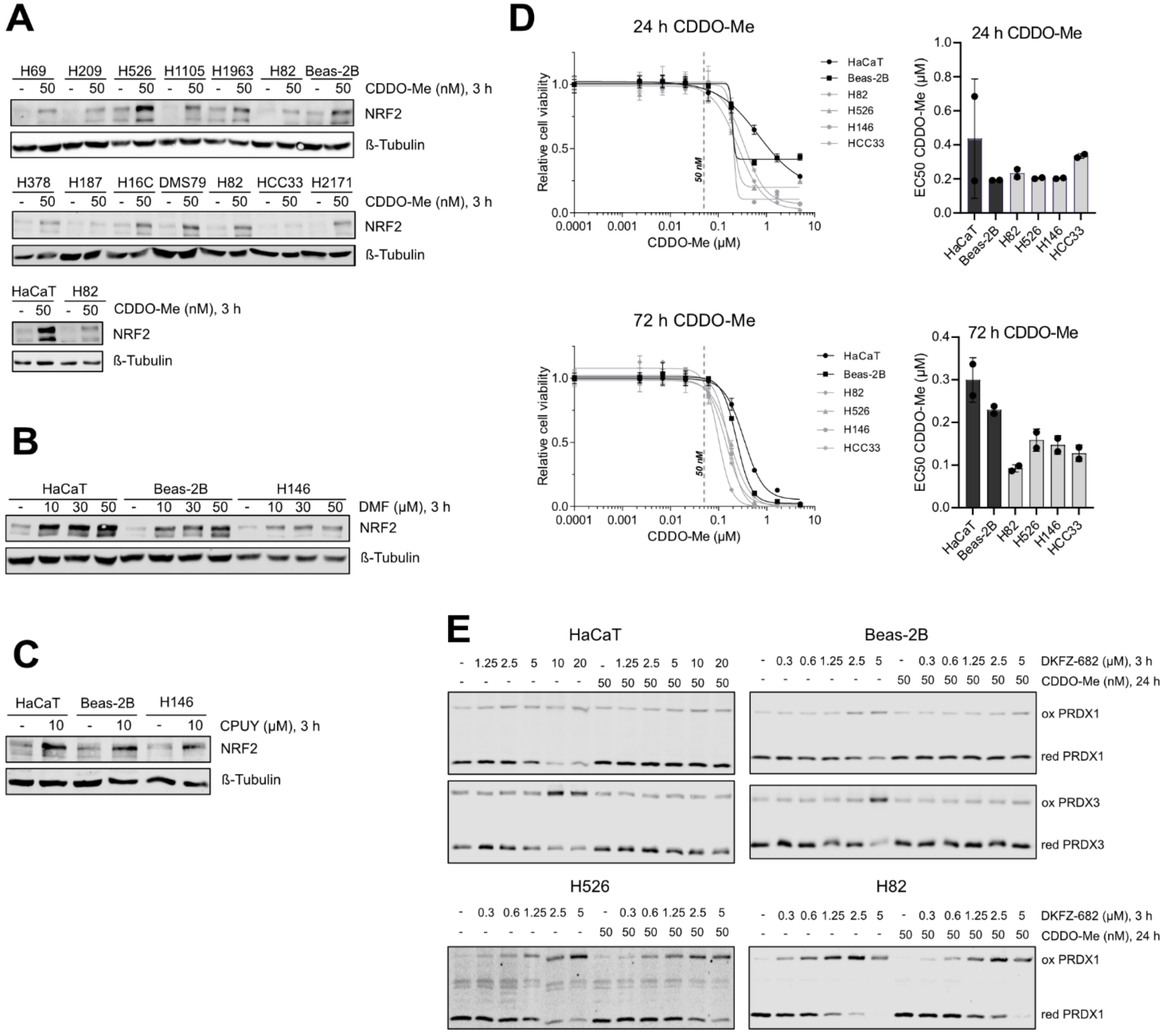

Figure S8: SCLC cell lines remain vulnerable to the cytotoxic effects of drugs, despite their high NRF2 protein level induced by CDDO-Me. (A-C) The cells were treated with DMSO (control), CDDO-Me, dimethyl fumarate (DMF) or CPUY192018 (CPUY) for 3 h and the protein level of NRF2 was analyzed by immunoblotting. The blots are representative of up to 6 independent experiments. **(D)** Cytotoxicity of CDDO-Me. Cells were treated with the indicated concentrations of CDDO-Me for 24 or 72 h. Cell viability

was assessed using CellTiter-Glo assay. Graphs on the left panel are representative of one experiment, each performed in biological triplicate. Quantitative results from independent experiments (n=2, mean ± SD) are summarized in the bar diagrams in the right panel. **(E)** Cell lines were pre-treated with CDDO-Me or DMSO for 24 h and then treated with the indicated concentration of DKFZ-682 for 3 h. Oxidized (ox) and reduced (red) levels of PRDX1 and PRDX3 proteins were analyzed by immunoblotting. The blots are representative of at least two independent experiments. Source data are provided as a Source Data file.

Figure S9

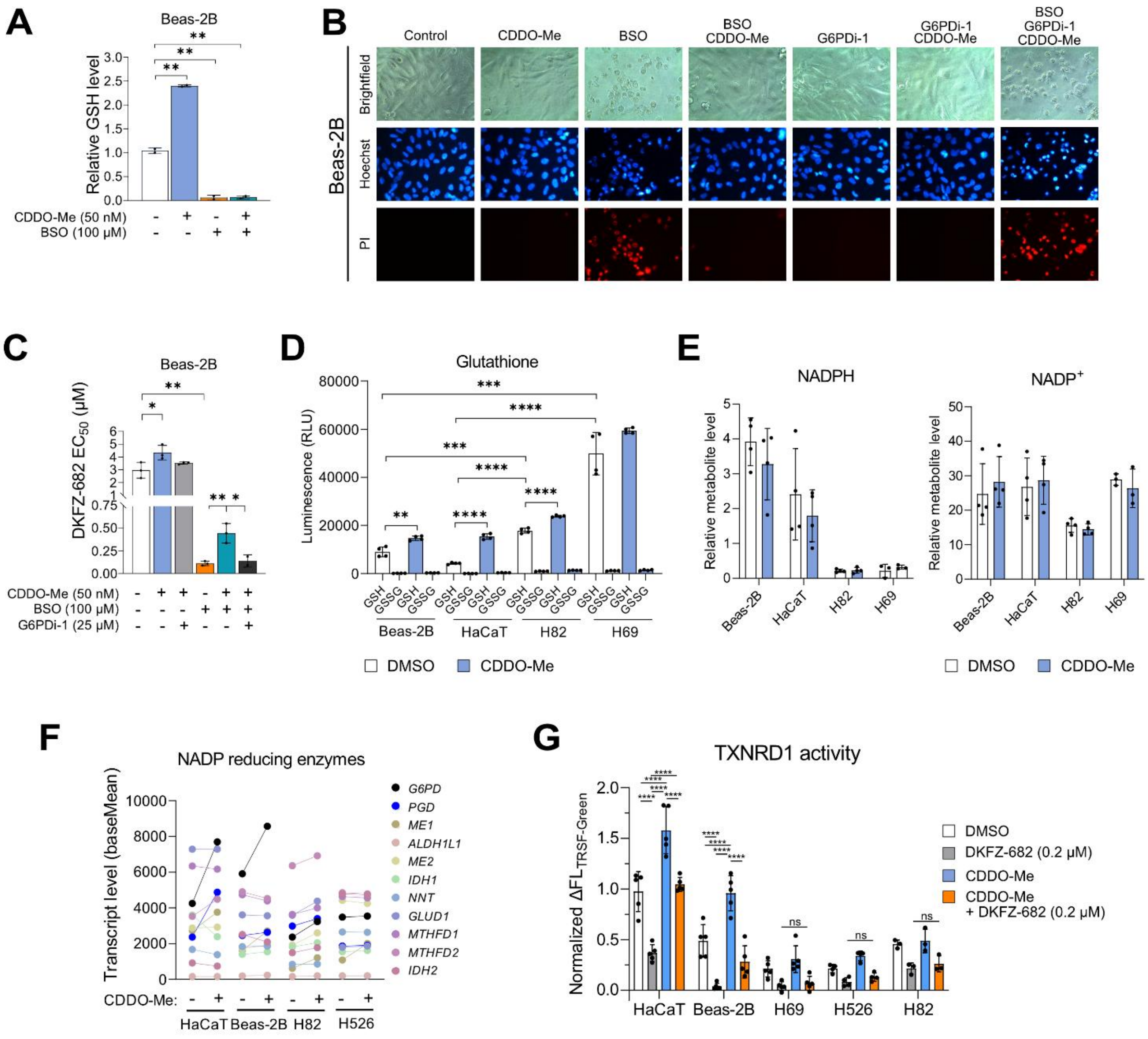

Figure S9: G6PD is involved in CDDO-Me-mediated protection of non-cancerous cells against ROS stress. (A-B) The cells were pre-treated with BSO (100 µM) for 24 h, followed by CDDO-Me (50 nM) alone or in combination with G6PDi-1 (25 µM) for another 24 h. **(A)** Endogenous levels of reduced glutathione were analyzed by reduced glutathione (GSH) assay (Abcam, ab235670; mean ± SD of two biological replicates; $^{**}p < 0.01$, two-tailed unpaired *t*-test). **(B)** Cells were co-stained with Hoechst 33342 to visualize all cells

and with propidium iodide (PI) to identify dead cells. The phase contrast and fluorescence microscopy images are representative of two independent experiments. **(C)** The cells were pre-treated with BSO for 24 h, followed by CDDO-Me alone or in combination with G6PDi-1 for another 24 h. Then the cells were treated with a concentration series of DKFZ-682 for 24 h. Cell viability was measured by the CellTiter-Glo assay. Data represent the mean ± SD of 3 independent experiments, each performed in biological triplicate (*$p < 0.05$, **$p < 0.01$, two-tailed unpaired *t*-test). **(D)** Cell lines were treated with DMSO (control) or CDDO-Me (50 nM) for 24 h. Endogenous levels of glutathione were analyzed in cell lysates using GSH/GSSG-Glo™ assay (Promega, V6611). The signal intensities were normalized to protein concentration of each sample. The results are mean ± SD of two independent experiments each performed in duplicates (**$p < 0.01$, ***$p < 0.001$, ****$p < 0.0001$, two-tailed unpaired *t*-test). **(E)** The endogenous levels of NADPH and $NADP^+$ were analyzed using metabolomic analysis (the processed raw data in **Supplementary Data 1**). The signal intensities were normalized to protein concentration of each sample (H69: $n = 3$; Beas-2B, HaCaT, H82: $n = 4$). **(F)** The transcript levels of enzymes involved in NADPH regeneration upon CDDO-Me treatment as determined by the expression profiling analysis ($n = 3$). **(G)** TXNRD1 activity was measured using the fluorescent dye TRFS-Green as a substrate in cells pre-incubated with CDDO-Me or DMSO as a control for 24 h. After re-seeding immediately before the measurement, DKFZ-682 was added to the final concentration of 0.2 µM or 20 µM, the latter serving to determine TXNRD1 unrelated background in each cell line. TXNRD1 activity was calculated as Δ fluorescence of TRFS-Green per s per µg (the protein concentration was determined using BCA assay in the cell suspension used for re-seeding). The activities in each experiment were normalized to the average activity of HaCaT and Beas-2B ("batch-normalization"). All cell lines were measured in independent experiments (H82: $n = 3$; H526, H69, Beas-2B, HaCaT: $n = 5$). Statistical analysis was performed using two-way ANOVA to evaluate main effects and interactions between cell lines and experimental treatments. Post-hoc pairwise comparisons were conducted using Tukey's test, with adjusted p-values reported for statistically significant intra-cell line comparisons (****$p < 0.0001$). Non-significant differences are specifically marked only for relevant treatment groups. Source data are provided as a Source Data file.

Figure S10

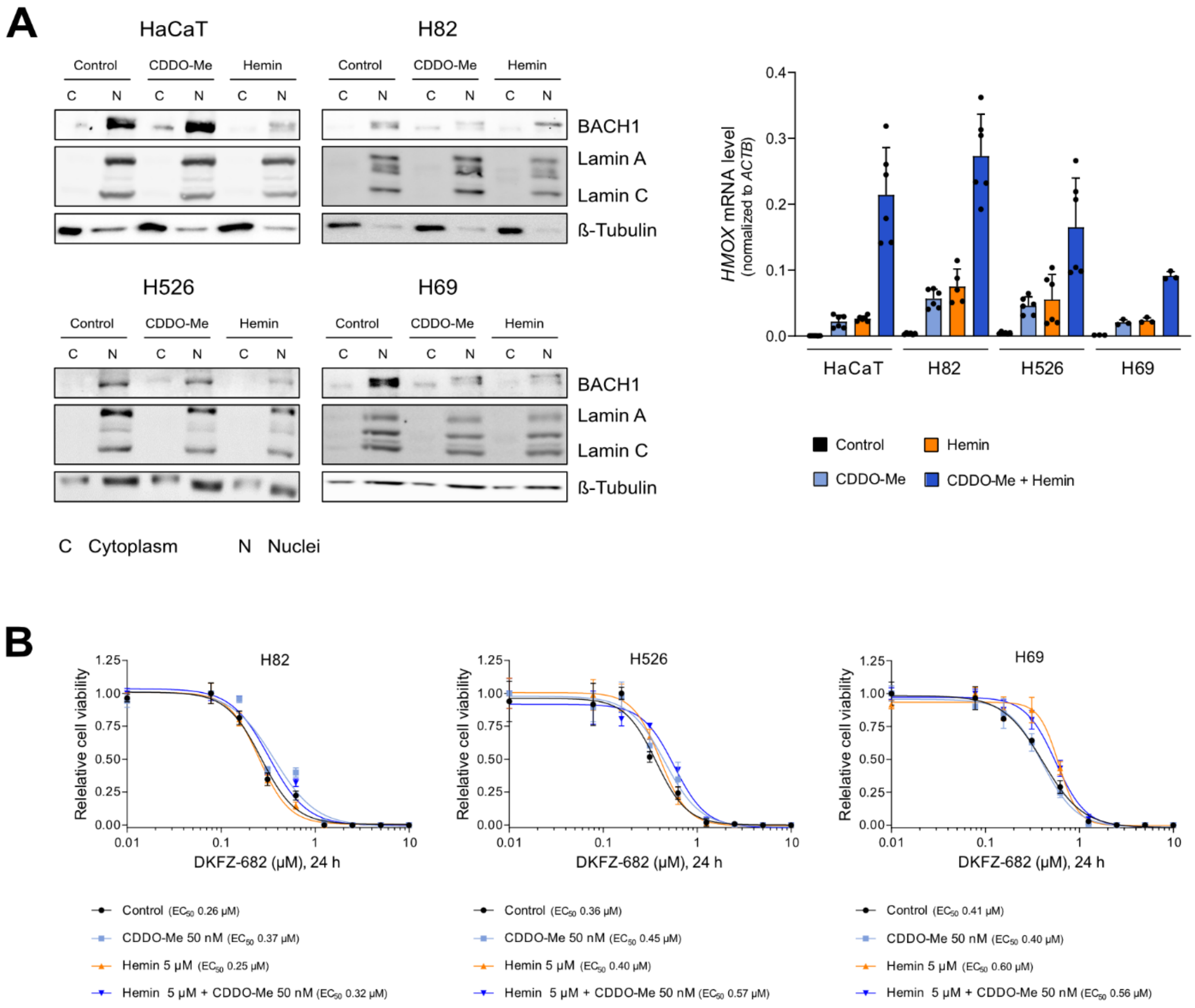

Figure S10: Inhibition of BACH1 does not affect sensitivity of SCLC to TXNRD1-targeting drug. (A) The indicated cell lines were treated with DMSO (control), CDDO-Me (50 nM) or hemin (10 µM) for 6 h. The protein levels of BACH1 (left panel) were analyzed in the cytoplasmic (C) and nuclear (N) fraction by immunoblotting. Lamin A/C and β-Tubulin served as controls for the nuclear and cytoplasmic sub-compartments, respectively. The blots are representative of two independent experiments. The level of *HMOX* (right panel), an indicator of BACH1 inhibition, was analysed by qPCR. The results (mean ± SD) are representative of two independent experiments, each performed in biological triplicates. **(B)** The cells were pre-treated with DMSO (control), CDDO-Me (50 nM) with/or hemin (5 µM) for 24 h. After that, a concentration series of DKFZ-682 was added for another 24 h. The cell viability was quantified by the

CellTiter-Glo assay. The results are representative of two independent experiments, each performed in biological triplicates. Source data are provided as a Source Data file.

Figure S11

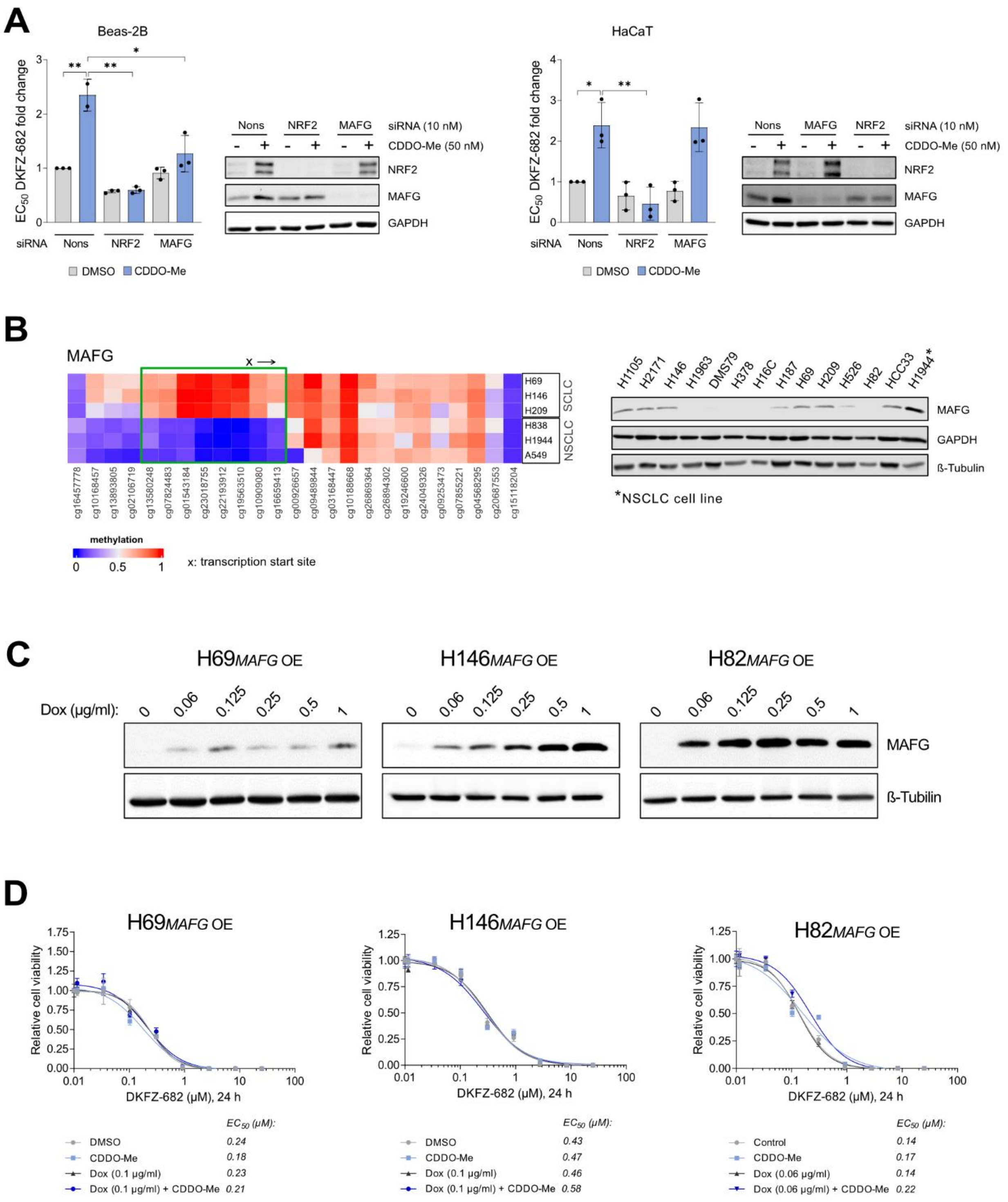

Figure S11: MAFG is essential for CDDO-Me inducible protection of Beas-2B against of cytotoxic effect of TXNRD-1 inhibition. (A) Cells were either treated with nonsense (Nons) siRNA or siRNA against *NRF2*

or *MAFG*. The next day, cells were trypsinized and 5,000 cells were seeded into the wells of a 96-well plate. After 24 h, cells were treated with DMSO (control) or CDDO-Me (50 nM). After additional 24 h, cells were treated with a range of concentrations of DKFZ-682 for 24 h. Cell viability was assessed using CellTiter-Glo assay. The graph is representative of independent experiments (n = 3, mean ± SD; *$p < 0.05$, **$p < 0.01$ two-tailed unpaired t-test) each performed in biological triplicates. EC_{50} fold change data are normalized to Nons. NRF2 and MAFG protein expression was analysed by immunoblotting. Representative western blots are shown.

Increased promoter methylation of *MAFG* gene in SCLC. (B) The heatmap, showing the methylation status of genomic DNA (left panel; 0 – no methylation, 1 – high methylation) was analysed using the Infinium MethylationEPIC BeadChips (Illumina). The protein level of MAFG (right panel) in total cell extracts from 13 SCLC cell lines was analyzed by immunoblotting (n = 2). High MAFG expression in drug resistant H1944 NSCLC cell line was used as a reference.

Combined NRF2 induction by CDDO-Me and overexpression of MAFG in SCLC cells does not lead to a desensitization of cells to TXNRD1 inhibition. (C) Cells with a tet-inducible MAFG expression construct were treated with doxycycline (Dox) for 24 h to increase MAFG expression. The protein level of MAFG was analyzed by immunoblotting. **(D)** Cells were pre-treated with Dox and/or CDDO-Me (50 nM) for 24 h to increase MAFG and NRF2 expression respectively. After that, a concentration series of DKFZ-682 was added for another 24 h and cell viability was measured by the CellTiter-Glo assay. The graph is representative of two independent experiments each performed in biological triplicate.

Source data are provided as a Source Data file.

Figure S12

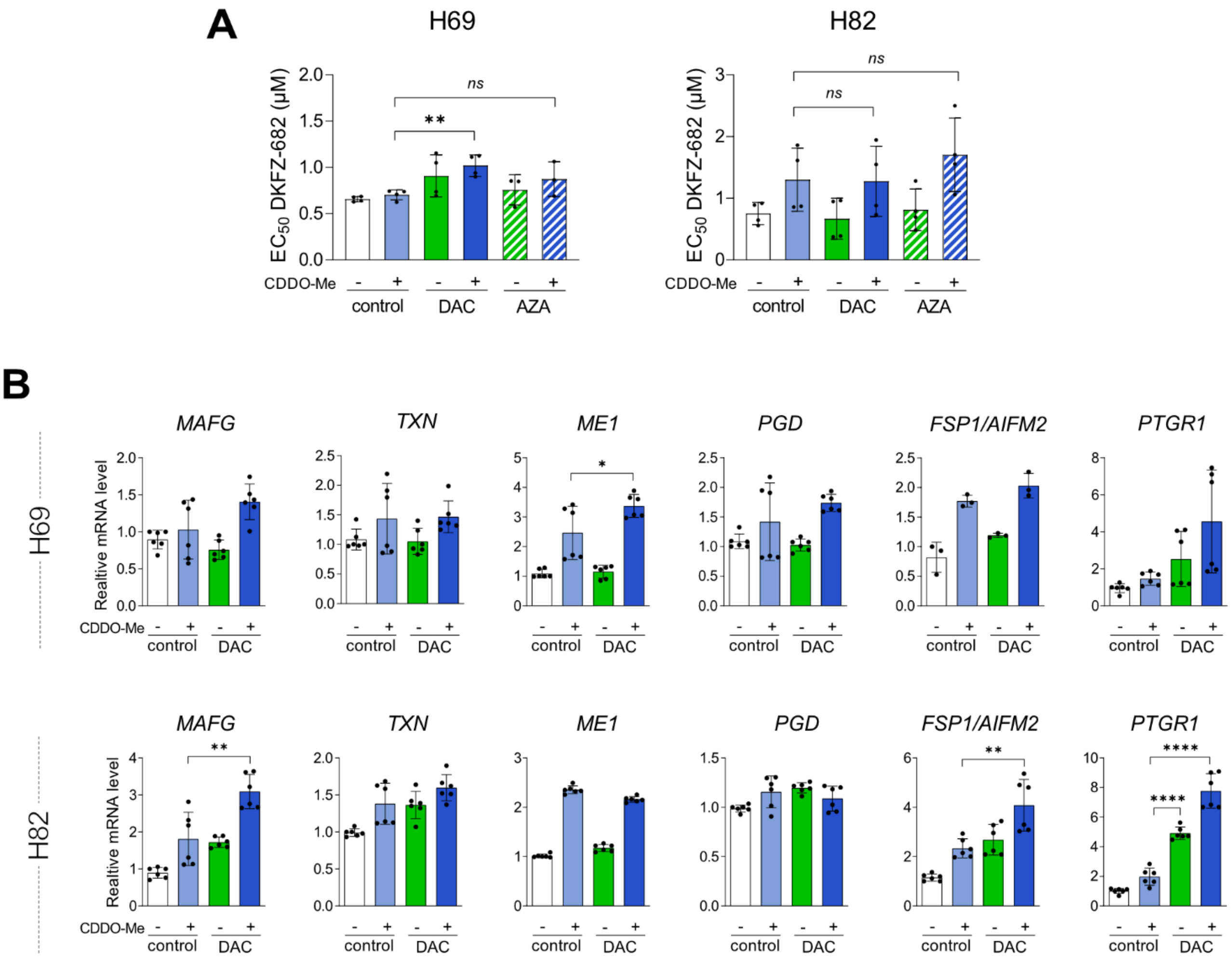

Figure S12: Combined Effect of pre-treatment with DAC and CDDO-Me on cell sensitivity to TXNRD inhibition and on ACB gene expression. (A, B) H69 and H82 cell lines were treated with decitabine (DAC, 0.5 µM) or azacitidine (AZA, 2.5 µM) three times over one week (on days one, three, and five). Afterward, the medium was replaced with DAC/AZA-free medium. **(A)** Cells were seeded in a 96-well plate and pre-treated with or without CDDO-Me (50 nM). After 24 hours, the cells were exposed to a concentration series of DKFZ-682 for 24 hours, and cell viability was measured using the CellTiter-Glo assay. Data represent the mean ± SD of at least three independent experiments, each performed in biological triplicate (**$p < 0.01$, two-tailed unpaired *t*-test). **(B)** Cells were treated with CDDO-Me (50 nM) for 6 h and levels of MAFG and ACB transcripts were determined by qPCR (Control without CDDO-Me was set to 1; relative data represent mean ± SD of two independent experiments each performed in triplicate; *ns*,

not significant, *$p < 0.05$, **$p < 0.01$, ****$p < 0.0001$, two-tailed unpaired *t*-test). Source data are provided as a Source Data file.

Figure S13

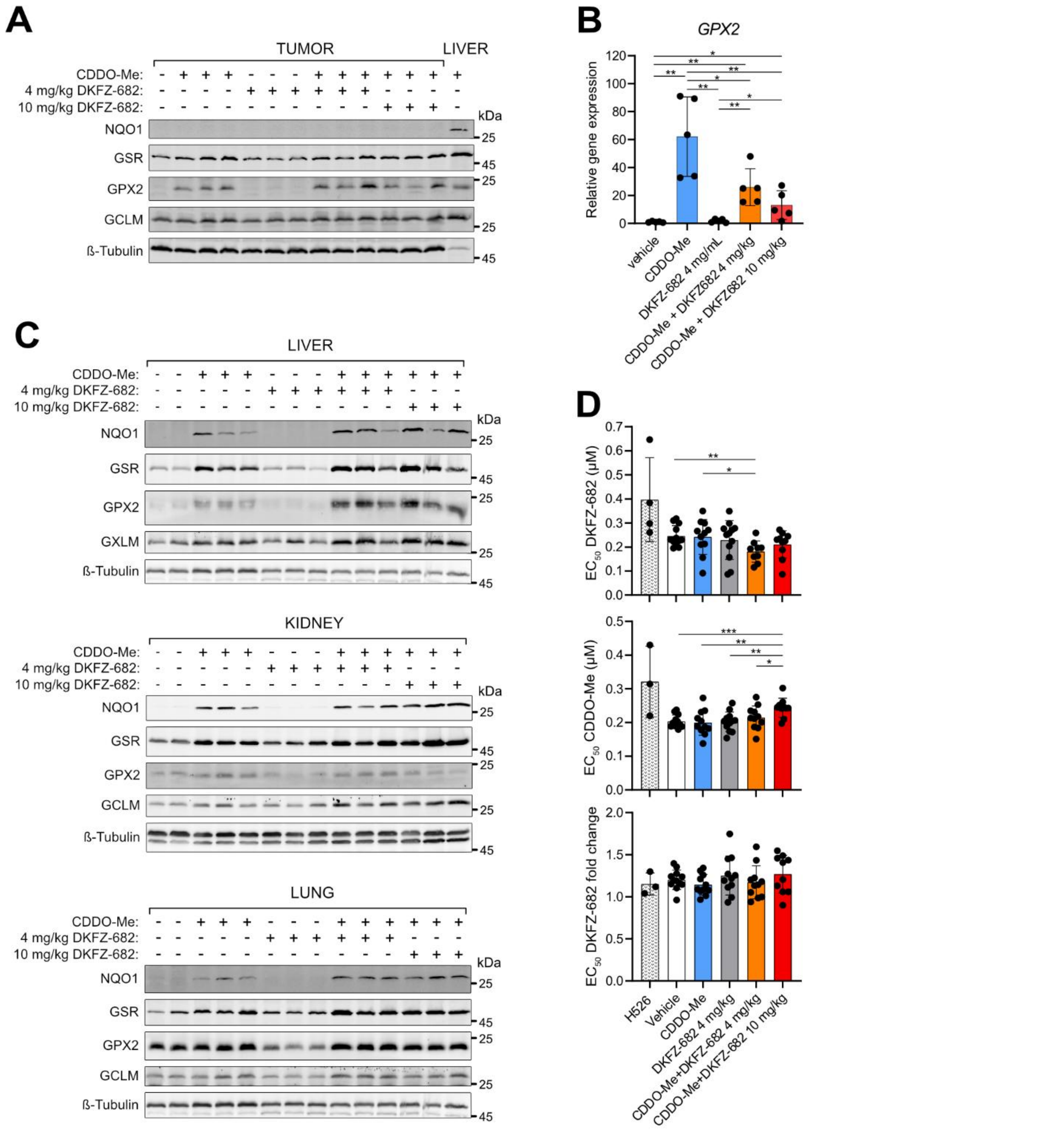

Figure S13: Impact of CDDO-Me pre-treatment on NRF2 target gene expression and cytotoxicity to the drug. (A–C) The effect of 4 and 10 mg/kg DKFZ-682 on the protein and RNA expression of NRF2 response

genes in tumors **(A, B)** and mouse tissues **(C)**, which were harvested when the humane endpoint was reached for each animal**.** The induction of NRF2 target was investigated by immunoblotting. Representative western blots are shown **(A, C)**. *GPX2* transcript levels were determined by qPCR in tumors of 5 animals from each treatment group (one-way ANOVA $p < 0.0001$; *$p < 0.05$, **$p < 0.01$ two-tailed unpaired t-test) **(B)**. **(D)** The sensitivity to DKFZ-682 and CDDO-Me was tested in explanted tumors. Tumor cells were treated with a concentration series of DKFZ-682 or CDDO-Me for 24 h and cell viability was quantified using CellTiter-Glo assay. Each dot represents a tumor from one mouse measured in a triplicate. Next, tumor cells were pre-treated with CDDO-Me (50 nM) for 24 h and then the concentration series of DKFZ-682 was applied. The fold change of EC_{50} for DKFZ-682 upon CDDO-Me compared to vehicle pre-treated cells was calculated (one-way ANOVA $p < 0.0001$; *$p < 0.05$, **$p < 0.01$, ***$p < 0.001$ two-tailed unpaired t-test). Non-significant differences between treatment groups are not specifically marked. Source data are provided as a Source Data file.

Figure S14

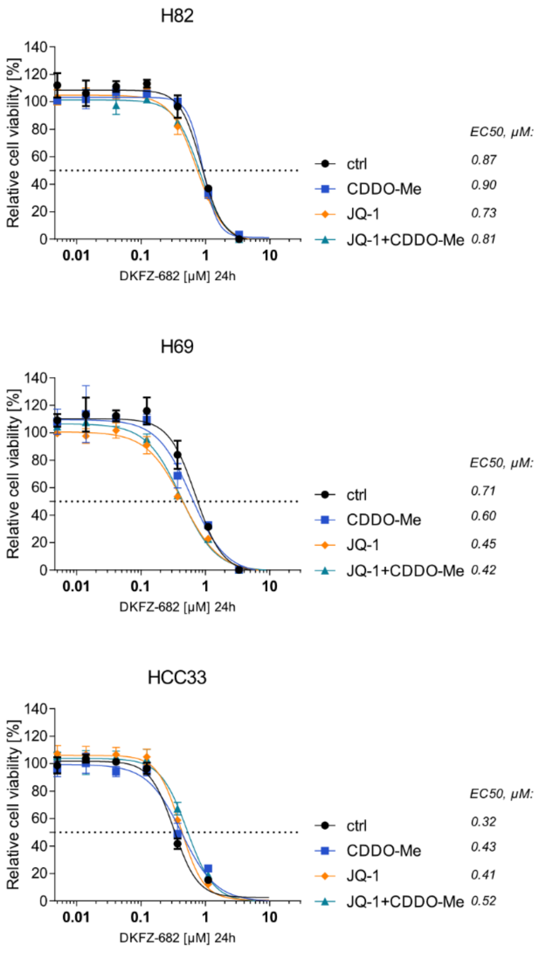

Figure S14: Inactivation of BRD4 does not enhance cell resistance to ROS inducing drug. SCLC cell lines were pre-treated with CDDO-Me (50 nM) and 1 µM JQ-1 or a combination of both. After 24 h, a concentration series of DKFZ-682 was added for another 24 h and the cell viability was measured by the CellTiter-Glo assay. The graphs are representative of two independent experiments each performed in triplicate. Source data are provided as a Source Data file.

Figure S15

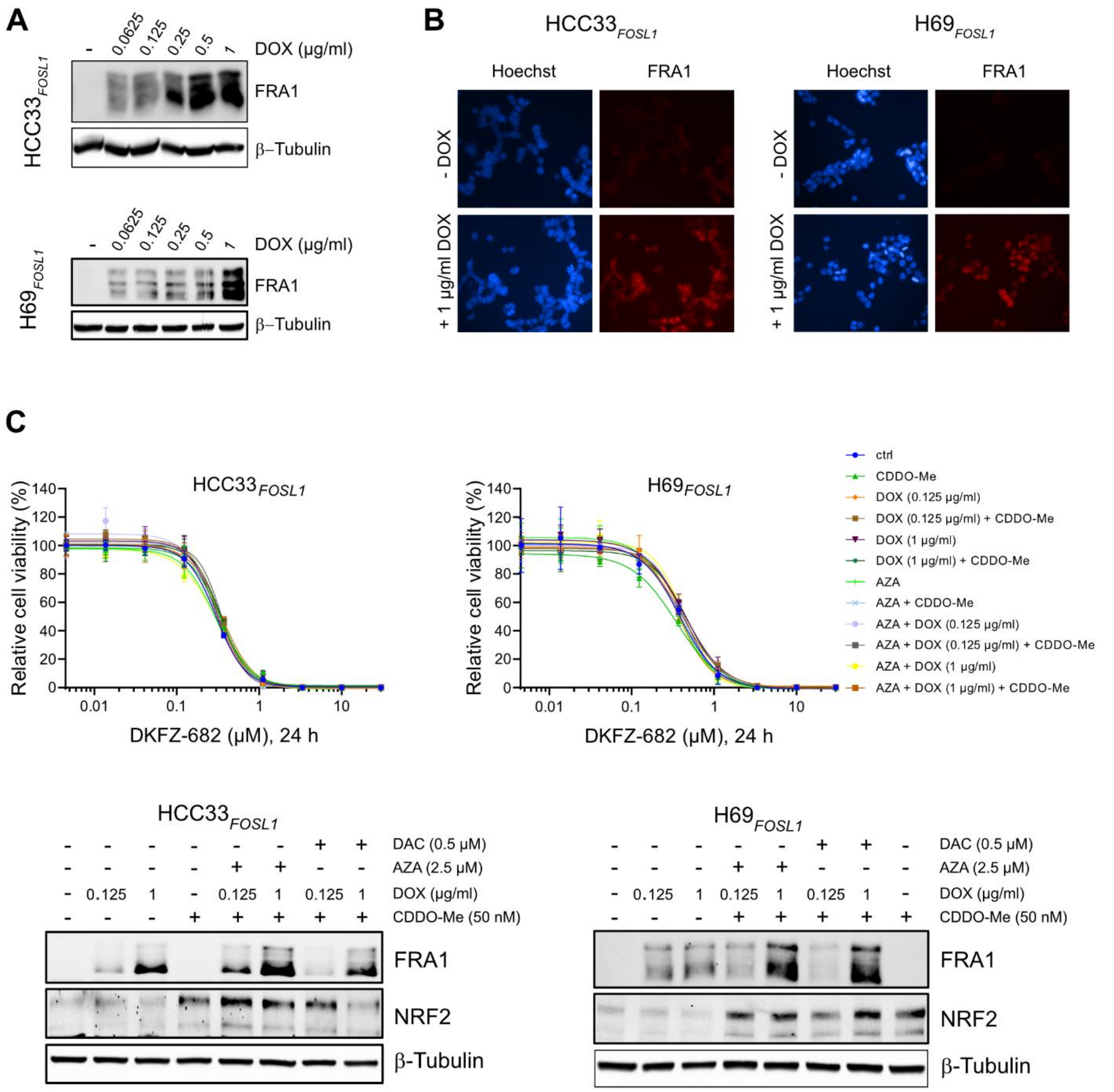

Figure S15: Combined NRF2 induction by CDDO-Me and overexpression of *FOSL1* in SCLC cells does not lead to a desensitization of cells to TXNRD1 inhibition. (A, B) HCC33$_{FOSL1}$ and H69$_{FOSL1}$ cells with a tet-inducible *FOSL1* expression construct were treated with doxycycline (DOX, 0.125 or 1 µg/mL) for 24 h to increase *FOSL1* expression. FRA1 expression was verified using immunoblotting (**A**) and IF (**B**). **(C)** HCC33$_{FOSL1}$ and H69$_{FOSL1}$ cells were treated with decitabine (DAC, 0.5 µM) or azacitidine (AZA, 2.5 µM) three times over one week (on days one, three, and five). Afterward, the medium was replaced with DAC/AZA-free medium. Cells were seeded in a 96-well plate and pre-treated with DOX and/or CDDO-Me

(50 nM) for 24 h to increase FRA1 and NRF2 expression respectively. Then the cells were treated with a concentration series of DKFZ-682 for 24 h and cell viability was measured by the CellTiter-Glo assay. The graph is representative of two independent experiments each performed in triplicate. The expression of FRA1 and NRF2 was verified using immunoblotting. Source data are provided as a Source Data file.

Supplementary Table S1: Chemical compounds

Chemical compound	Company	Catalog No
1S,3R-RSL 3 (RSL3)	Sigma-Aldrich	SML2234
Auranofin	Sigma-Aldrich	A6733
Azacitidine (AZA)	Selleckchem	S1782
Beta-Cyclodextrin sulfobutyl ether sodium salt	Biosynth	OC15979
2-Hydroxy ß-Cyclodextrin	Sigma-Aldrich	332607
Buthionine sulfoximine (BSO)	Sigma-Aldrich	B2640
CDDO-Me	Tocris	6646
Cycloheximid (CHX)	Santa Cruz Biotechnology	SC-3508B
Cisplatin	Santa Cruz Biotechnology	SC-200896A
CPUY192018 (CPUY)	Biotrend	AOB9974-1
Decitabine (DAC)	Hölzel Diagnostica	TMO-T1508
Diamide (DA)	Sigma-Aldrich	D3648
Dimethylsulfoxide (DMSO)	Sigma-Aldrich	D2650
Deferoxamine (DFO)	Sigma-Aldrich	D9533
DKFZ-608	produced by AG Gunkel/Miller, DKFZ	
DKFZ-682	produced by AG Gunkel/Miller, DKFZ	
Dimethyl fumarate (DMF)	Sigma-Aldrich	242926
Dithiothreitol (DTT)	Serva	20711.02
Ferrostatin-1 (Fer-1)	Sigma-Aldrich	SML0583
G6PDi-1	Sigma-Aldrich	SML2980
Hoechst 33342	Cayman chemical	15547
JQ1	Sigma-Aldrich	SML1524
ML210	Sigma-Aldrich	SML0521
N-Acetyl-L-cystein	Sigma-Aldrich	A9165
Necrostatin (Nec-1)	Merck	480065
Olaparib (AZD2281)	Cell Signaling	93852
Propidium Iodide (PI)	Sigma-Aldrich	P4864
Puromycin	Sigma-Aldrich	P9620
Sodium aurothiomalate	Fisher Scientific	11344707
Tetraethyldithiuram disulphide	Sigma-Aldrich	86720
Z-VAD-FMK	Adooq Bioscience	A12373
TRFS green	produced by AG Gunkel/Miller, DKFZ, as described by Zhang et al.[10]	

Supplementary Table S2: Primers

Cloning ORFs into pLIX-403

Target	Primer sequence	
SalI-MAFG	AAAAGTCGACGCCACCATGACGACCCCCAATAAAGGA	fwd
MAFG-AvrII	AAAACCTAGGCTACGATCGGGCATCCGT	rev
SalI-FOSL1	AAAAGTCGACGCCACCATGTTCCGAGACTTCGGGGAAC	fwd
FOSL1-AvrII	AAAACCTAGGCTACAAAGCGAGGAGGGTTGGAG	rev

Supplementary Table S3: Primary and secondary antibodies

Antigen	Product / Company	Species	Dilution WB
AIFM2/FSP1	20886-1-AP/Proteintech	rabbit	1:1000
AKR1C3	A6229/ Sigma-Aldrich	mouse	1:500
ATF4	11815/ Cell Signaling	rabbit	1:1000
BACH1	NBP1-71814/ Novus	rabbit	1:2000
FRA1	ab252421/ Abcam	rabbit	1:1000
FTL	ab69090/ Abcam	rabbit	1:1000
GAPDH	sc-365062/ Santa Cruz	mouse	1:1000
GCLC	ab207777/ Abcam	rabbit	1:1000
GCLM	HPA023696/ Sigma-Aldrich	rabbit	1:100
GPX2	ab137431/ Abcam	rabbit	1:1000
GPX4	ab125066-1001/ Abcam	rabbit	1:1000
GSR	ab124995/ Abcam	rabbit	1:1000
Lamin A/C	2032/ Cell Signaling	rabbit	1:1000
MAFG	ab154318/ Abcam	rabbit	1:1000
ME1	ab97445/ Abcam	rabbit	1:1000
NQO1	ab28947/ Abcam	mouse	1:1000
NRF2	ab62352/ Abcam	rabbit	1:1000
PRDX1	8499/ Cell Signaling	rabbit	1:1000
PRDX3	ab128953/ Abcam	rabbit	1:2000
SLC7A11	12691/ Cell Signaling	rabbit	1:1000
TXN (TRX1)	ab133524/ Abcam	rabbit	1:10000
TXNRD1 (TRXR1)	ab16820/ Abcam	rabbit	1:1000
β-Tubulin	T0198/ Sigma-Aldrich	mouse	1:1000
IRDye 680LT anti-mouse IgG	926-68022/ LI-COR	donkey	1:5000
RDye 680LT anti-rabbit IgG	926-68023/ LI-COR	donkey	1:5000
StarBright Blue 520 anti-rabbit IgG	12005869/ Biorad	goat	1:3000
StarBright Blue 700 anti-rabbit IgG	12004162/ Biorad	goat	1:3000
StarBright Blue 520 anti-mouse IgG	12005866/ Biorad	goat	1:3000
StarBright Blue 700 anti-mouse IgG	12004159/ Biorad	goat	1:3000

Supplementary Table S4: qPCR primers

Human

Target	Primer forward 5´- 3´	Primer reverse 3´- 5´
AKR1C3	CCGAAGCAAGATTGCAGATGGC	GTGAGTTTTCCAAGGCTGGTCG
ACTB	CCACCATGTACCCTGGCATT	CGCTCAGGAGGAGCAATGAT
AIFM2/FSP1	GACTCCTTCCACCACAATGTGG	CAGCACCATCTGGTTCTTCAGG
G6PD	CTGTTCCGTGAGGACCAGATCT	TGAAGGTGAGGATAACGCAGGC
GAPDH	TGCGACTTCAACAGCAACTC	CTTGCTCAGTGTCCTTGCTG
GPX2	GACTTCACCCAGCTCAACGA	CCCCAGGACGGACATACTTG
HMOX1	CCAGGCAGAGAATGCTGAGTTC	AAGACTGGGCTCTCCTTGTTGC
MAFG	TGACGACCCCCAATAAAGGAAA	TCACCAGCTCCTCATCCGT
ME1	GGAGTTGCTCTTGGTGTTGTGG	GGATAAAGCCGACCCTCTTCCA
NRF2 (NFE2L2)	CACATCCAGTCAGAAACCAGTGG	GGAATGTCTGCGCCAAAAGCTG
NQO1	CCTGCCATTCTGAAAGGCTGGT	GTGGTGATGGAAAGCACTGCCT
PGD	GTTCCAAGACACCGATGGCAAAC	CACCGAGCAAAGACAGCTTCTC
PTGR1	GGAAAAGCTGCTGACAGAGTGG	CACTGTTTCTCCACCCTTCACAC
TXN (TRX)	GTTGACTTCTCAGCCACGTG	TCACCCACCTTTTGTCCCTT

Supplementary Table S5: LC gradient for metabolomics

Time (min)	Flow (mL/min)	%A	%B
**0**	0,400	5	95
**0,5**	0,400	5	95
**0,51**	0,350	5	95
**5**	0,350	90	10
**5,1**	0,300	90	10
**5,2**	0,300	100	0
**10**	0,300	100	0
**10,3**	0,400	5	95
**15**	0,400	5	95

References

1 Nishizawa, H., Yamanaka, M. & Igarashi, K. Ferroptosis: regulation by competition between NRF2 and BACH1 and propagation of the death signal. *FEBS J* **290**, 1688-1704 (2023). https://doi.org:10.1111/febs.16382

2 Warnatz, H. J. *et al.* The BTB and CNC homology 1 (BACH1) target genes are involved in the oxidative stress response and in control of the cell cycle. *J Biol Chem* **286**, 23521-23532 (2011). https://doi.org:10.1074/jbc.M111.220178

3 Casares, L. *et al.* The synthetic triterpenoids CDDO-TFEA and CDDO-Me, but not CDDO, promote nuclear exclusion of BACH1 impairing its activity. *Redox Biol* **51**, 102291 (2022). https://doi.org:10.1016/j.redox.2022.102291

4 Marro, S. *et al.* Heme controls ferroportin1 (FPN1) transcription involving Bach1, Nrf2 and a MARE/ARE sequence motif at position -7007 of the FPN1 promoter. *Haematologica* **95**, 1261-1268 (2010). https://doi.org:10.3324/haematol.2009.020123

5 Sun, J. *et al.* Hemoprotein Bach1 regulates enhancer availability of heme oxygenase-1 gene. *EMBO J* **21**, 5216-5224 (2002). https://doi.org:10.1093/emboj/cdf516.

6 Hirotsu, Y. *et al.* Nrf2-MafG heterodimers contribute globally to antioxidant and metabolic networks. *Nucleic Acids Res* **40**, 10228-10239 (2012). https://doi.org:10.1093/nar/gks827

7 Liu, S., Pi, J. & Zhang, Q. Signal amplification in the KEAP1-NRF2-ARE antioxidant response pathway. *Redox Biol* **54**, 102389 (2022). https://doi.org:10.1016/j.redox.2022.102389

8 Dhakshinamoorthy, S. & Jaiswal, A. K. Small maf (MafG and MafK) proteins negatively regulate antioxidant response element-mediated expression and antioxidant induction of the NAD(P)H:Quinone oxidoreductase1 gene. *J Biol Chem* **275**, 40134-40141 (2000). https://doi.org:10.1074/jbc.M003531200

9 Dull, T. *et al.* A third-generation lentivirus vector with a conditional packaging system. *J Virol* **72**, 8463-8471 (1998). https://doi.org:10.1128/JVI.72.11.8463-8471.1998

10 Zhang, L. *et al.* Highly selective off-on fluorescent probe for imaging thioredoxin reductase in living cells. *J Am Chem Soc* **136**, 226-233 (2014). https://doi.org:10.1021/ja408792k

11 Morgan, B. *et al.* Real-time monitoring of basal H2O2 levels with peroxiredoxin-based probes. *Nat Chem Biol* **12**, 437-443 (2016). https://doi.org:10.1038/nchembio.2067

12 Rost, L. M., Shafaei, A., Fuchino, K. & Bruheim, P. Zwitterionic HILIC tandem mass spectrometry with isotope dilution for rapid, sensitive and robust quantification of pyridine nucleotides in biological extracts. *J. Chromatogr. B Analyt. Technol. Biomed. Life Sci.* **1144**, 122078 (2020). https://doi.org:10.1016/j.jchromb.2020.122078

13 Cai, L. *et al.* Cell-autonomous immune gene expression is repressed in pulmonary neuroendocrine cells and small cell lung cancer. *Commun Biol* **4**, 314 (2021). https://doi.org:10.1038/s42003-021-01842-7

14 Morgen, M., Fabrowski, P., Amtmann, E., Gunkel, N. & Miller, A. K. Inclusion Complexes of Gold(I)-Dithiocarbamates with beta-Cyclodextrin: A Journey from Drug Repurposing towards Drug Discovery. *Chem. Eur. J.* **27**, 12156-12165 (2021). https://doi.org:10.1002/chem.202101366

15 Cai, L. *et al.* The small cell lung cancer neuroendocrine transdifferentiation explorer. *bioRxiv*, 2022.2008.2001.502252 (2022). https://doi.org:10.1101/2022.08.01.502252

16 Tlemsani, C. *et al.* SCLC-CellMiner: A Resource for Small Cell Lung Cancer Cell Line Genomics and Pharmacology Based on Genomic Signatures. *Cell Rep* **33**, 108296 (2020). https://doi.org:10.1016/j.celrep.2020.108296

17 Samarin, J. *et al.* Low level of antioxidant capacity biomarkers but not target overexpression predicts vulnerability to ROS-inducing drugs. *Redox Biol* **62**, 102639 (2023). https://doi.org:10.1016/j.redox.2023.102639

18 Setsukinai, K., Urano, Y., Kakinuma, K., Majima, H. J. & Nagano, T. Development of novel fluorescence probes that can reliably detect reactive oxygen species and distinguish specific species. *J Biol Chem* **278**, 3170-3175 (2003). https://doi.org:10.1074/jbc.M209264200

19 Luo, Z. *et al.* Fluorescent real-time quantitative measurements of intracellular peroxynitrite generation and inhibition. *Anal Biochem* **520**, 44-48 (2017). https://doi.org:10.1016/j.ab.2017.01.001

20 Kojima, H. *et al.* Fluorescent Indicators for Imaging Nitric Oxide Production. *Angew Chem Int Ed Engl* **38**, 3209-3212 (1999). https://doi.org:10.1002/(sici)1521-3773(19991102)38:21<3209::aid-anie3209>3.0.co;2-6
