# Supplementary material for: Differential KEAP1/NRF2 mediated signaling widens the therapeutic window of redox-targeting drugs in SCLC therapy
Source: Nat Commun. 2026 Apr 12;17:3435. doi: 10.1038/s41467-026-71608-4 (PMC13076645; doi:10.1038/s41467-026-71608-4)
Supplement: Supplementary file 2 — Description of Additional Supplementary Files [file 41467_2026_71608_MOESM2_ESM.pdf]

Description of Additional Supplementary Files

Supplementary Data 1: Metabolomics data processed to quantify NADPH and NADP+ levels in cell pellets.
