# Supplementary material for: Differential KEAP1/NRF2 mediated signaling widens the therapeutic window of redox-targeting drugs in SCLC therapy
Source: Nat Commun. 2026 Apr 12;17:3435. doi: 10.1038/s41467-026-71608-4 (PMC13076645; doi:10.1038/s41467-026-71608-4)
Supplement: Supplementary file 4 — Reporting Summary [file 41467_2026_71608_MOESM4_ESM.pdf]

nature portfolio

Corresponding author(s): Nikolas Gunkel

Last updated by author(s): Mar 9, 2026

Reporting Summary

Nature Portfolio wishes to improve the reproducibility of the work that we publish. This form provides structure for consistency and transparency in reporting. For further information on Nature Portfolio policies, see our Editorial Policies and the Editorial Policy Checklist.

Statistics

For all statistical analyses, confirm that the following items are present in the figure legend, table legend, main text, or Methods section.

n/a	Confirmed	
☐	☒	The exact sample size (*n*) for each experimental group/condition, given as a discrete number and unit of measurement
☐	☒	A statement on whether measurements were taken from distinct samples or whether the same sample was measured repeatedly
☐	☒	The statistical test(s) used AND whether they are one- or two-sided<br>*Only common tests should be described solely by name; describe more complex techniques in the Methods section.*
☒	☐	A description of all covariates tested
☐	☒	A description of any assumptions or corrections, such as tests of normality and adjustment for multiple comparisons
☐	☒	A full description of the statistical parameters including central tendency (e.g. means) or other basic estimates (e.g. regression coefficient) AND variation (e.g. standard deviation) or associated estimates of uncertainty (e.g. confidence intervals)
☐	☒	For null hypothesis testing, the test statistic (e.g. *F*, *t*, *r*) with confidence intervals, effect sizes, degrees of freedom and *P* value noted<br>*Give P values as exact values whenever suitable.*
☒	☐	For Bayesian analysis, information on the choice of priors and Markov chain Monte Carlo settings
☒	☐	For hierarchical and complex designs, identification of the appropriate level for tests and full reporting of outcomes
☐	☒	Estimates of effect sizes (e.g. Cohen's *d*, Pearson's *r*), indicating how they were calculated

Our web collection on statistics for biologists contains articles on many of the points above.

Software and code

Policy information about availability of computer code

Data collection	Measurement of fluorescence and luminescence was done with a FLUOstar OPTIMA or CLARIOstar (BMG LABTECH). FACS analysis was performed with Guava easyCyte 14HT flow cytometer (Cytek Biosciences) or BD LSR Fortessa (BD Biosciences). qPCR was performed on the Roche Lightcycler 480 system. Microarrays were scanned with Affymetrix GeneArray Scanner3000. For metabolite analysis, an ACQUITY I-class PLUS UPLC system (Waters) coupled to a QTRAP 6500+ (AB SCIEX) mass spectrometer with electrospray ionization source was used and data were acquired using Analyst 1.7.2 (AB SCIEX).
Data analysis	Heatmaps visualising cell death pathway component expression were generated using RStudio version 1.1.456 and gplots package version 3.1.1 and RColorBrewer package version 1.1-2 were used. Tumor growth and body weight were analyzed using linear mixed-effects models with restricted maximum likelihood estimation (REML) in R v4.3.1 (lme4 v1.1-35), post-hoc comparisons of simple treatment effects were conducted using the emmeans package (v1.9.0) with multivariate t-distribution (mvt) adjustment for multiple comparisons. CellTiter-Glo and CellTiter-Blue assays were analysed using GraphPad Prism 9. MTT assay was analysed using Origin 9.1 (OriginLab). The IHC staining in human samples was evaluated using the QuPath software (v. 0.1.2). Microscopic images were processed using Las Ez (version 3.1.0). Western blotting data were quantified using Image Studio Lite (Li-Cor) or Image Lab software (Bio-Rad). Data analysis for DNA methylation was performed with the Illumina's GenomeStudio 2011.1 (Modul M Version 1.9.0). Flow cytometry data were analyzed using FlowJo. The data were analyzed with a commercial software called JMP Genomics, version 7, from SAS. Gene expression profiling was performed using arrays of human Clariom S type from Affymetrix. A Custom CDF Version 22 with Entrez based gene definitions was used to annotate the arrays. The Raw fluorescence intensity values were normalized applying quantile normalization, RMA background correction and Medianpolish Probeset Summary. For the analysis of the known reference transcriptome assembly, RNA sequencing data were processed automatically via the One Touch Pipeline (OTP) (Resinger et al. 2017). Alignment program: STAR Version 2.5.3a Merging/duplication marking

program: Sambamba Version 0.6.5; SAMtools program: Version 1.6; Pipeline: RNAseqWorkflow:1.3.0). The metabolite data were processed using the OS software suite 2.0.0 (AB SCIEX).

For manuscripts utilizing custom algorithms or software that are central to the research but not yet described in published literature, software must be made available to editors and reviewers. We strongly encourage code deposition in a community repository (e.g. GitHub). See the Nature Portfolio guidelines for submitting code & software for further information.

Data

Policy information about availability of data

All manuscripts must include a data availability statement. This statement should provide the following information, where applicable:

- Accession codes, unique identifiers, or web links for publicly available datasets
- A description of any restrictions on data availability
- For clinical datasets or third party data, please ensure that the statement adheres to our policy

All data supporting the findings in this study are available from the corresponding author upon reasonable request. RNAseq and microarray data are available in the GEO repository under the accession number GSE280643 [https://www.ncbi.nlm.nih.gov/geo/query/acc.cgi?acc=GSE280643]. The primary data underlying the graphs are provided in the Source Data File.
The publicly available data used in this study are available in the European Genome-Phenome Archive under the primary accession number EGAS00001000334 [https://ega-archive.org/studies/EGAS00001000334] as well as in George, J., et al., Comprehensive genomic profiles of small cell lung cancer. Nature, 2015. 524(7563): p. 47-53 and Rudin, C.M., et al., Comprehensive genomic analysis identifies SOX2 as a frequently amplified gene in small-cell lung cancer. Nat Genet, 2012. 44(10): p. 1111-6.

Research involving human participants, their data, or biological material

Policy information about studies with human participants or human data. See also policy information about sex, gender (identity/presentation), and sexual orientation and race, ethnicity and racism.

Reporting on sex and gender

Use the terms sex (biological attribute) and gender (shaped by social and cultural circumstances) carefully in order to avoid confusing both terms. Indicate if findings apply to only one sex or gender; describe whether sex and gender were considered in study design; whether sex and/or gender was determined based on self-reporting or assigned and methods used.
Provide in the source data disaggregated sex and gender data, where this information has been collected, and if consent has been obtained for sharing of individual-level data; provide overall numbers in this Reporting Summary. Please state if this information has not been collected.
Report sex- and gender-based analyses where performed, justify reasons for lack of sex- and gender-based analysis.

Reporting on race, ethnicity, or other socially relevant groupings

Please specify the socially constructed or socially relevant categorization variable(s) used in your manuscript and explain why they were used. Please note that such variables should not be used as proxies for other socially constructed/relevant variables (for example, race or ethnicity should not be used as a proxy for socioeconomic status).
Provide clear definitions of the relevant terms used, how they were provided (by the participants/respondents, the researchers, or third parties), and the method(s) used to classify people into the different categories (e.g. self-report, census or administrative data, social media data, etc.)
Please provide details about how you controlled for confounding variables in your analyses.

Population characteristics

Describe the covariate-relevant population characteristics of the human research participants (e.g. age, genotypic information, past and current diagnosis and treatment categories). If you filled out the behavioural & social sciences study design questions and have nothing to add here, write "See above."

Recruitment

Describe how participants were recruited. Outline any potential self-selection bias or other biases that may be present and how these are likely to impact results.

Ethics oversight

Identify the organization(s) that approved the study protocol.

Note that full information on the approval of the study protocol must also be provided in the manuscript.

Field-specific reporting

Please select the one below that is the best fit for your research. If you are not sure, read the appropriate sections before making your selection.

☒ Life sciences ☐ Behavioural & social sciences ☐ Ecological, evolutionary & environmental sciences

For a reference copy of the document with all sections, see nature.com/documents/nr-reporting-summary-flat.pdf

Life sciences study design

All studies must disclose on these points even when the disclosure is negative.

Sample size

Sample size calculations were not conducted for cell culture experiments and at least 3 biological replicate experiments were tested. For animal experiments, sample size was calculated with the help of a biostatistician. Assumptions for the power analysis were as follows: Alpha error 5%; Beta error 20%.

Data exclusions | No replicates were excluded.

Replication | Most of the experiments were performed at least 3 times independently. Exact replicate numbers are stated in the figure legends.

Randomization | In vitro treatments of cells and animal experiments were performed randomized..

Blinding | Except for randomization, we did not actively apply blinding measures in our experiments. We tried to avoid bias by performing in vitro experiments in several biological replicates by different researchers in the lab.

Reporting for specific materials, systems and methods

We require information from authors about some types of materials, experimental systems and methods used in many studies. Here, indicate whether each material, system or method listed is relevant to your study. If you are not sure if a list item applies to your research, read the appropriate section before selecting a response.

Materials & experimental systems

n/a	Involved in the study
☐	☒ Antibodies
☐	☒ Eukaryotic cell lines
☒	☐ Palaeontology and archaeology
☐	☒ Animals and other organisms
☒	☐ Clinical data
☒	☐ Dual use research of concern
☒	☐ Plants

Methods

n/a	Involved in the study
☒	☐ ChIP-seq
☐	☒ Flow cytometry
☒	☐ MRI-based neuroimaging

Antibodies

Antibodies used

All antibodies used in this study are listed in Supplementary Table 3.
Antigen Product / Company Species Dilution WB
AIF 4642/ Cell Signaling rabbit 1:1000
AIFM2/FSP1 20886-1-AP/Proteintech rabbit 1:1000
AKR1C3 A6229/ Sigma-Aldrich mouse 1:500
BACH1 NBP1-71814/ Novus rabbit 1:2000
FTL ab69090/ Abcam rabbit 1:1000
GAPDH sc-365062/ Santa Cruz mouse 1:1000
GCLC ab207777/ Abcam rabbit 1:1000
GCLM HPA023696/ Sigma-Aldrich rabbit 1:100
GPX2 ab137431/ Abcam rabbit 1:1000
GSR ab124995/ Abcam rabbit 1:1000
Lamin A/C 2032/ Cell Signaling rabbit 1:1000
MAFG ab154318/ Abcam rabbit 1:1000
ME1 ab97445/ Abcam rabbit 1:1000
NQO1 ab28947/ Abcam mouse 1:1000
NRF2 ab62352/ Abcam rabbit 1:1000
PRDX1 8499/ Cell Signaling rabbit 1:1000
PRDX3 ab128953/ Abcam rabbit 1:2000
SLC7A11 12691/ Cell Signaling rabbit 1:1000
TXN (TRX1) ab133524/ Abcam rabbit 1:10000
TXNRD1 (TRXR1) ab16820/ Abcam rabbit 1:1000
β-Tubulin T0198/ Sigma-Aldrich mouse 1:1000
IRDye 680LT anti-mouse IgG 926-68022/ LI-COR donkey 1:5000
RDye 680LT anti-rabbit IgG 926-68023/ LI-COR donkey 1:5000
StarBright Blue 520 anti-rabbit IgG 12005869/ Biorad goat 1:3000
StarBright Blue 700 anti-rabbit IgG 12004162/ Biorad goat 1:3000
StarBright Blue 520 anti-mouse IgG 12005866/ Biorad goat 1:3000
StarBright Blue 700 anti-mouse IgG 12004159/ Biorad goat 1:3000

Validation | All antibodies used in this study are commercially available, widely utilized, and validated on the respective company websites.

Eukaryotic cell lines

Policy information about cell lines and Sex and Gender in Research

Cell line source(s)

ATCC:
NCI-H1944 CRL-5907
NCI-H69 HTB-119

NCI-H82 HTB-175
NCI-H526 CRL-5811
NCI-H209 HTB-172
NCI-H1105 CRL-5856
NCI-H187 CRL-5804
DMS79 CRL-2049
NCI-H146 HTB-173
NCI-H2171 CRL-5929
NCI-H1963 CRL-5982
NCI-H378 CRL-5808
A549 CRM-CCL-185
NCI-H838 CRL-5844
Beas-2B CRL-3588
DSMZ:
HCC33 487
SCLC-16HC (SCLC-16H) [Bepler, G., et al., Markers and characteristics of human SCLC cell lines. Neuroendocrine markers, classical tumor markers, and chromosomal characteristics of permanent human small cell lung cancer cell lines. J Cancer Res Clin Oncol, 1987. 113(3): p. 253-9.]
Addex Bio:
HaCaT T0020001

Authentication

Cell lines were authenticated using Multiplex Cell Authentication by Multiplexion (Heidelberg) as described in [Castro, F., et al., High-throughput SNP-based authentication of human cell lines. Int J Cancer, 2013. 132(2): p. 308-14]

Mycoplasma contamination

Cell lines were tested negative for mycoplasma contamination (Eurofins Genomics).

Commonly misidentified lines
(See ICLAC register)

SCLC-16H

Animals and other research organisms

Policy information about studies involving animals; ARRIVE guidelines recommended for reporting animal research, and Sex and Gender in Research

Laboratory animals

The study involved female mice (6–7 weeks old) of the nude strain BALB/c (BALB/cAnNCrl, Charles River) and female mice (8-10 weeks old) of the NSG (NOD.Cg- Prkdcscid Il2rgtm1Wjl/SzJ; DKFZ Heidelberg).

Wild animals

The study did not involve wild animals.

Reporting on sex

Female mice were chosen for the study because the tumor-bearing animals are randomized at the start of the experiment and grouped together in cages. This is only easily done with females, as male animals are difficult to regroup later due to their territorial behavior.

Field-collected samples

The study did not involve field-collected samples.

Ethics oversight

All studies involving mice were conducted in compliance with German Cancer Research Center guidelines and approved by the governmental review board of the state of Baden-Württemberg, Karlsruhe District Council, under authorization no. G-191/16, G-259/18, and G-176/19, according to German legal regulations.

Note that full information on the approval of the study protocol must also be provided in the manuscript.

Plants

Seed stocks

Report on the source of all seed stocks or other plant material used. If applicable, state the seed stock centre and catalogue number. If plant specimens were collected from the field, describe the collection location, date and sampling procedures.

Novel plant genotypes

Describe the methods by which all novel plant genotypes were produced. This includes those generated by transgenic approaches, gene editing, chemical/radiation-based mutagenesis and hybridization. For transgenic lines, describe the transformation method, the number of independent lines analyzed and the generation upon which experiments were performed. For gene-edited lines, describe the editor used, the endogenous sequence targeted for editing, the targeting guide RNA sequence (if applicable) and how the editor was applied.

Authentication

Describe any authentication procedures for each seed stock used or novel genotype generated. Describe any experiments used to assess the effect of a mutation and, where applicable, how potential secondary effects (e.g. second site T-DNA insertions, mosiacism, off-target gene editing) were examined.

Flow Cytometry

Plots

Confirm that:

☒ The axis labels state the marker and fluorochrome used (e.g. CD4-FITC).

☒ The axis scales are clearly visible. Include numbers along axes only for bottom left plot of group (a 'group' is an analysis of identical markers).

☒ All plots are contour plots with outliers or pseudocolor plots.

☒ A numerical value for number of cells or percentage (with statistics) is provided.

Methodology

Sample preparation	Lipid peroxidation was analyzed in cells stained with Bodipy 581/591 C11 (Invitrogen). For detection of ROS/RNS level cells were treated with 5 µM CM-H2DCFDA (Invitrogen, Thermo Scientific, Cat No C6827), 1:250 OxiVision Green peroxide sensor (AAT Bioquest, Cat No. 11506, powder was solved in 200 µL DMSO), 5 µM DAF-FM Diacetate (Invitrogen, Thermo Scientific, Cat No. D23844) and 1:400 DAX-J2™ PON Green (AAT Bioquest, Cat No. 16317).
Instrument	FACS analysis was performed with Guava easyCyte 14HT flow cytometer (Cytek Biosciences) or BD LSR Fortessa (BD Biosciences).
Software	Flow cytometry data were analyzed using FlowJo.
Cell population abundance	No sorting was performed.
Gating strategy	Live cells were gated based on FSC-A/FSC-H and single cells were gated based on BLUE-V-A/BLUE-V-H (Guava) or SSC-A/SSC-W (BD LSR Fortessa). Single cell population was used to generate histograms in the channel suitable for each dye. Median intensities of histograms of several replicate experiments were determined and plotted as bar graphs to evaluate statistics.

☒ Tick this box to confirm that a figure exemplifying the gating strategy is provided in the Supplementary Information.
